# Probing Intracellular Yeast Metabolism With Deuterium Magnetic Resonance Spectroscopy

**DOI:** 10.1002/nbm.70121

**Published:** 2025-09-02

**Authors:** Fatima Anum, Charbel Assaf, Farhad Haj Mohamad, Maria Anikeeva, Arne Brahms, Jyotirmoy Dey, Simon Kaltenberger, Eric Beitz, Lina Welz, Victoria Annis, Manuel van Gemmeren, Simon Duckett, Jan‐Bernd Hövener, Andrey N. Pravdivtsev

**Affiliations:** ^1^ Section Biomedical Imaging, Molecular Imaging North Competence Center, Department of Radiology and Neuroradiology University Hospital Schleswig‐Holstein, Kiel University Kiel Germany; ^2^ Otto Diels Institute of Organic Chemistry Kiel University Kiel Germany; ^3^ Pharmaceutical Institute Kiel University Kiel Germany; ^4^ Institute of Clinical Molecular Biology, Department of Internal Medicine I University Medical Center Schleswig‐Holstein, Kiel University Kiel Germany; ^5^ Centre for Hyperpolarization in Magnetic Resonance University of York York UK

**Keywords:** deuterium labeling, DMRS, intracellular metabolism, metabolomics, noninvasive, osmotic stress, real‐time flux measurement, *Saccharomyces cerevisiae*

## Abstract

Metabolomics provides snapshots of states of metabolites under specific conditions, with nuclear magnetic resonance (NMR) being one of the few noninvasive techniques. However, when applied to intact cells (e.g., yeast or mammalian cells) or tissues, traditional ^1^H NMR often suffers from overlapping signals from numerous metabolites and intracellular macromolecules such as proteins. To address this, we employed deuterium‐labeled tracers that do not suffer from background interference and streamline targeted flux analysis. Deuterium magnetic resonance spectroscopy (DMRS) enables rapid, noninvasive measurement of metabolic flux without specialized equipment. In our study, we first measured *T*_1_, *T*_2_, and chemical shifts for 26 deuterium‐labeled compounds in phosphate‐buffered saline: parameters functional for optimal DMRS settings. Among the 26 deuterated compounds tested with food‐grade baker's yeast ( *Saccharomyces cerevisiae* ) as an easily accessible model solution, we observed and tracked the real‐time consumption of pyruvate, glucose, fumarate, acetone, and nicotinamide. We redirected yeast metabolism by (i) varying concentrations of added pyruvate and (ii) osmotic pressure by changing buffer density. This study underscores DMRS's potential as a robust, versatile tool for dissecting metabolic transformations exemplified here with the convenient yeast cell systems active for hundreds of minutes under typical NMR observation conditions.

## Graphical abstract

Deuterium magnetic resonance spectroscopy (DMRS) provides a straightforward, noninvasive method for monitoring real‐time metabolic flux. We determined the T1, T2, and chemical shifts for 26 deuterium‐labeled compounds in phosphate‐buffered saline. DMRS showed the consumption of pyruvate, glucose, fumarate, acetone, and nicotinamide, and let us test metabolic flexibility by changing the supply of pyruvate and osmotic pressure. This work demonstrates DMRS as a flexible tool for dissecting metabolic transformations in intact cells and proves yeast to be a convenient test system.

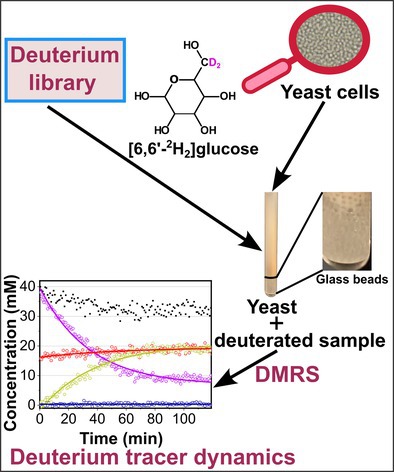

**Abbreviations**
Ac‐*d*_2_
: [2,2‐^2^H_2_]acetate
Ac‐*d*_3_
: [2,2,2‐^2^H_3_]acetate
Ace‐*d*_6_
: [U‐^2^H_6_]acetone
Acetal‐*d*_2_
: [2,2‐^2^H_2_]acetaldehyde
Acetal‐*d*_3_
: [2,2,2‐^2^H_3_]acetaldehyde
Acetal‐*d*_4_
: [1,2,2,2‐^2^H_4_]acetaldehyde
Aceto‐*d*_2_
: [1,1‐^2^H_2_]acetoin
ACN‐*d*_3_
: [2,2,2‐^2^H_3_]acetonitrile
ADH: alcohol dehydrogenase
Ala‐*d*_3_
: [3,3,3‐^2^H_3_]alanine
ALD: aldehyde dehydrogenase
BD‐*d*_2_
: [1,1‐^2^H_2_]butanediol
BDH: butanediol dehydrogenase
DMRI: deuterium magnetic resonance imaging
DMRS: deuterium magnetic resonance spectroscopy
DMSO‐*d*_6_
: [U‐^2^H_6_]dimethyl sulfoxide
Eth‐*d*_2_
: [2,2‐^2^H_2_]ethanol
Eth‐*d*_3_
: [2,2,2‐^2^H_3_]ethanol
Eth‐*d*_6_
: [U‐^2^H_6_]ethanol
ETX‐*d*_2_
: [4,4′‐^2^H_2_]ethosuximide
Form‐*d*
: [1‐^2^H]formate
Fum‐*d*_2_
: [2,3‐^2^H_2_]fumarate
Glc‐*d*_2_
: [6,6′‐^2^H_2_]glucose
Glc‐*d*_7_
: [U‐^2^H_7_]glucose
Glut‐*d*_2_
: [4,4‐^2^H_2_]glutamate
Gly‐*d*_8_
: [U‐^2^H_8_]glycerol
HDO: [^2^H]water
Hyd‐Acetal‐*d*_4_
: [1,2,2,2‐^2^H_4_]hydrated‐acetaldehyde
Lact‐*d*_3_
: [3,3,3‐^2^H_3_]lactate
Mal‐*d*_2_
: [2,3‐^2^H_2_]malate
Meth‐*d*_4_
: [U‐^2^H_4_]methanol
NA‐*d*_4_
: [2,4,5,6‐^2^H_4_]nicotinic acid
NAM‐*d*_4_
: [2,4,5,6‐^2^H_4_]nicotinamide
PBS: phosphate‐buffered saline
PDC: pyruvate decarboxylase
Pnc1: nicotinamidase
Prop‐*d*_6_
: [1,1,1,3,3,3‐^2^H_6_]propan‐2‐ol
Pyr‐*d*_2_
: [3,3‐^2^H_2_]pyruvate
Pyr‐*d*_3_
: [3,3,3‐^2^H_3_]pyruvate
Ser‐*d*_3_
: [2,3,3′‐^2^H_3_]serine
Suc‐*d*_4_
: [2,2,3,3‐^2^H_4_]succinate
TMSP‐*d*_4_
: [2,2,3,3‐^2^H_4_]trimethylsilylpropionic acid
Trp‐*d*_5_
: [2,4,5,6,7‐^2^H_5_]tryptophan

## 1 Introduction

Metabolomics provides valuable insights into the systemic identification and quantification of small molecules, including sugars, amino acids, short fatty acids, organic acids, and steroids [1], providing detailed information about the biochemical pathways, cellular processes, and physiological states [2, 3]. Nuclear magnetic resonance (NMR) spectroscopy and mass spectrometry coupled with gas chromatography (GC–MS) or liquid chromatography (LC–MS) [4] are the most common tools for studying metabolomics. GC–MS/LC–MS are highly sensitive but destructive for the studied samples, often require special sample preparation, and hence do not allow for studying living systems or longitudinal studies of one sample [5]. In contrast, NMR allows for noninvasive, quantitative metabolite profiling both in vitro and in vivo, statically and dynamically; however, it is 10–100 times less sensitive than MS‐based techniques [6, 7, 8, 9].

Still, conventional ^1^H NMR typically offers rich metabolomic information from cell lysates or other processed cell extracts in a single snapshot rather than capturing dynamic metabolic changes over time; hence, a different sample is required for each time point [10, 11, 12, 13]. In contrast, when analyzing intact cells or unprocessed samples, the tiny signals of metabolites considerably overlap and are masked with the signals from lipids and proteins [14], making quantitative real‐time analysis complicated or impossible.

In parallel to ^1^H NMR, it is feasible to use non‐proton nuclei, such as ^31^P from endogenous molecules like adenosine triphosphate (ATP) [2, 15, 16, 17], to observe cellular energy states by detecting changes in phosphate levels [18, 19, 20, 21].

Alternatively, labeled tracers with NMR‐sensitive isotopes such as ^2^H, ^13^C, ^15^N, or ^19^F can be administered and monitored across different reactions noninvasively in vivo. The naturally occurring background signal of such nuclei is typically low compared with the added labeled tracer that eases signal assignment and analysis. For example, ^13^C enriched glucose [22], acetate [23], pyruvate [24], choline [25], and fumarate [26] have been extensively used in metabolic studies of microbes [27], plant cells [28, 29], mammalian cells [30], and in humans [31] to provide valuable information on metabolic flux.

In many cases, the low sensitivity of the isotopes was improved by transferring the polarization from or to protons [22]. An alternative method to increase the signal in NMR is to use nuclear spin hyperpolarization [32, 33, 34, 35]. Hyperpolarization boosts the NMR signal of the active nuclei by several orders of magnitude, allowing for real‐time metabolic measurements of living cells with a few seconds of resolution [36, 37, 38]. However, this modality has significant disadvantages as it requires specialized, expensive equipment (a polarizer), making it costly and allowing only a short observation window of 1 or 2 min during hyperpolarization signal decay [39, 40, 41, 42, 43].

Deuterium magnetic resonance imaging (DMRI) and spectroscopy (DMRS) were recently rediscovered [44, 45, 46] and have shown strong potential for metabolic imaging in humans [47] and animals [48]. In contrast to hyperpolarized MR, the only equipment needed here is a deuterium‐compatible NMR or MRI system, offering a real‐time, noninvasive assessment of metabolism. In addition to clinical applications, DMRS offers a robust method for studying biochemical processes in vitro [49, 50], enabling tracking of selected cellular metabolism and its enzymatic kinetics noninvasively and in real‐time. Deuterium‐labeled tracers are typically more accessible than ^13^C or ^15^N‐labeled analogs or can be synthesized on demand [51, 52].

Although deuterium has about 6.5 times lower gyromagnetic ratio than proton [53], resulting in much lower sensitivity, this can be partially compensated by its typically rapid relaxation time, *T*_1_, of ~100 ms, which enables rapid signal averaging without saturation. The low natural abundance of deuterium, around 0.0156% [54, 55], results in little background signal, allowing clear detection of ^2^H‐enriched exogenous substrates. The only background signal comes from water, which can be used as an internal reference for quantification [56].

To date, only a few deuterium‐labeled tracers have been tested with DMRS: [1‐^2^H], [6,6′‐^2^H_2_], and [U‐^2^H_7_]glucose [47, 57, 58, 59, 60, 61, 62]; [3,3,3‐^2^H_3_]pyruvate [61]; [2,2,2‐^2^H_3_]acetate [47]; [^2^H_9_]choline [63]; 3‐O‐CD_3_‐glucose [64]; [2,3‐^2^H_2_]fumarate [49, 65, 66]; and [2,3,3′‐^2^H_3_]serine [67]. Nevertheless, many important biologically relevant tracers remain unreported despite the simplicity and versatility of DMRS. Furthermore, the signal‐to‐noise ratio (SNR) of most metabolites remains low—even at high field strengths—and extensive signal averaging is often required, limiting the number of traceable metabolic steps requiring systematic optimization of DMRS protocols.

We thus set out to investigate the magnetic resonance (MR) properties and metabolic behavior of 26 deuterated molecules (Table 1), chosen for their relevance to a broad range of metabolic pathways (e.g., the tricarboxylic acid cycle [TCA], glycolysis, amino acid metabolism, and salvage pathway). We identified their chemical shifts and *T*_1_ and *T*_2_ relaxation times, some of which were reported before at different magnetic fields [60], which are critical for maximizing DMRS sensitivity and optimizing the acquisition protocol.

This methodical testing helps find tracers with favorable NMR properties and robust metabolic turnover, which makes them suitable for future imaging applications. By creating a comprehensive reference dataset of DMRS‐visible tracers and their metabolites, we offer a foundational resource for designing DMRS and DMRI experiments across a range of biological models—from in vitro systems to preclinical and potentially clinical in vivo imaging—even in setups where lower scanner sensitivity may pose a challenge.

As for testing the metabolic profile of these tracers, we chose food‐grade baker's yeast as a model system ( *Saccharomyces cerevisiae* ), which was recently described in hyperpolarization‐enhanced metabolic studies [68, 69, 70]. Yeast offers a reproducible, genetically tractable platform. It is cost‐effective for evaluating MR tracer performance under controlled conditions. Its ease of cultivation and minimal infrastructure requirements make it particularly suitable for optimizing experimental parameters before translation to mammalian or clinical imaging, especially in laboratories lacking access to cell culture facilities, such as hospital radiology departments. As a single‐cell eukaryote, yeast shares key metabolic pathways with higher organisms [71, 72] and can be easily engineered for specific metabolic processes [73]. Such properties make yeast a practical system for tracer screening and protocol development. Hence, this study is a methodological foundation for applying and optimizing DMRI and DMRS in preclinical and clinical research by offering standardized tracer protocols and metabolic readouts.

Apart from functioning as a standard metabolic model, certain yeast strains [74, 75] possess considerable potential for being used to get insights into the biochemistry of human disorders. Examples include dysbiosis‐associated inflammatory bowel diseases (IBD) [76, 77], obesity [78], and diabetes [79]. Overgrowth *of Candida albicans* yeast strain has been shown to worsen intestinal inflammation by inducing Th17 immune responses and compromising the gut barrier [75, 80]. Yeasts are typically understudied in this context, even though they are relevant. This shows that more research is needed on their role in human health and disease.

Metabolic products were detected in six of the 22 tracers. To demonstrate DMRS' sensitivity to metabolic disruption of glycolysis and salvage pathway, we investigated the effect of supplementing pyruvate [68, 81] on the [6,6′‐^2^H_2_]glucose (Glc‐*d*_2_) and [2,4,5,6‐^2^H_4_]nicotinamide (NAM‐*d*_4_) metabolism and the impact of phosphate buffer (PB) concentrations on the fumarate‐to‐malate metabolism, highlighting DMRS utility in probing glycolysis, TCA cycle activity, and the nicotinamide adenine dinucleotide (NAD^+^) salvage process.

## 2 Methods

### 2.1 Materials

Eighteen deuterium‐labeled compounds were purchased and used without purification: [2,2,2‐^2^H_3_]acetate (Ac‐*d*_3_, DLM‐3126, Eurisotop), [1,2,2,2‐^2^H_4_]acetaldehyde (Acetal‐*d*_4_, 176,567, Sigma Aldrich), [U‐^2^H_6_]acetone (Ace‐*d*_6_, 44,863, Sigma Aldrich), [2,2,2‐^2^H_3_]acetonitrile (ACN‐*d*_3_, 00205, Deutero GmbH)), [U‐^2^H_6_]dimethyl sulfoxide (DMSO‐*d*_6_, D010F, Eurisotop), [U‐^2^H_6_]ethanol (Eth‐*d*_6_, 02702, Deutero GmbH), [1‐^2^H]formate (Form‐*d*, DLM‐1361, Eurisotop), [2,3‐^2^H_2_]fumarate (Fum‐*d*_2_, 778,370, Sigma Aldrich), [6,6′‐^2^H_2_]glucose (Glc‐*d*_2_, DLM‐349, Eurisotop), [U‐^2^H_7_]glucose (Glc‐*d*_7_, 55,203, Sigma Aldrich), [U‐^2^H_8_]glycerol (Gly‐*d*_8_, DLM‐558‐1, Eurisotop), [1,2,2,2‐^2^H_4_]hydrated‐acetaldehyde (Hyd‐Acetal‐*d*_4_, 176,567, Sigma Aldrich), [3,3,3‐^2^H_3_]lactate (Lact‐*d*_3_, DLM‐9071, Eurisotop), [U‐^2^H_4_]methanol (Meth‐*d*_4_, 151,947, Sigma Aldrich), [3,3,3‐^2^H_3_]pyruvate (Pyr‐*d*_3_, DLM‐6068, Eurisotop), [2,3,3′‐^2^H_3_]serine (Ser‐*d*_3_, DLM‐582, Eurisotop), [2,2,3,3‐^2^H_4_]succinate (Suc‐*d*_4_, 293,075, Sigma Aldrich), and [2,2,3,3‐^2^H_4_]trimethylsilylpropionic acid (TMSP‐*d*_4_, 00905, Deutero GmbH). Four deuterium‐labeled compounds, [2,4,5,6,7‐^2^H_5_]tryptophan (Trp‐*d*_5_), [3,3,3‐^2^H_3_]alanine (Ala‐*d*_3_), [2,4,5,6‐^2^H_4_]nicotinamide (NAM‐*d*_4_), and [4,4′‐^2^H_2_]ethosuximide (ETX‐*d*_2_), were synthesized in‐house. The synthesis of Trp‐*d*_5_ and Ala‐*d*_3_ is detailed in Figures S7a–j and S8a–j, respectively. NAM‐*d*_4_ and ETX‐*d*_2_ were synthesized according to protocols described in [82] and [83], with the corresponding procedures included in Supporting Information.

Food‐grade dried *S. cerevisiae* , commonly known as baker's yeast, was purchased from a commercial grocery store. The ingredients in the yeast include baker's yeast and emulsifier (sorbitan monostearate, E491). Products from everal manufacturers were tested, and the results were essentially the same. Here, only one product was used for consistency.

The phosphate‐buffered saline (PBS) used in this study was purchased as a ready‐to‐use solution (Gibco PBS 100 mM, pH 7.4, Catalog No. 70011044, Thermo Fisher Scientific, Waltham, MA, USA). The composition of this solution is outlined in the product specifications provided by the manufacturer.

### 2.2 NMR Tube Preparation

First, we added 0.15 g of 0.5 mm glass beads (Z250465, Sigma Aldrich), which occupy about 60 μL of the standard 5‐mm NMR tube volume. The purpose of adding the glass beads was to elevate the sample to the sensitive detection zone of the coil [69, 84], maximizing the signal strength and ensuring that the yeast always stayed in the sensitive area of the NMR. Afterward, 45 μL of PBS was added to soak the beads and prevent CO_2_ produced by the yeast from becoming trapped in the spaces between them.

### 2.3 Protocol for DMRS of *S. cerevisiae*

One gram of baker's yeast was suspended in 7 mL of deionized water within a 15‐mL Falcon tube. The suspension was then incubated for 10 min at 32°C (~305 K) on a water bath, with the tube shaken every 3 min to facilitate the release of carbon dioxide. Following incubation, 248 μL of the yeast suspension, containing approximately 4.216 × 10^8^ cells, was transferred to the preheated 5‐mm NMR tube containing 0.15 g of 0.5 mm glass beads and 45 μL of PBS. The tube was then immediately placed into a 400‐MHz NMR spectrometer (Bruker Avance NEO, WB) maintained at 310 K. A thin ETFE capillary tubing (1/32″ outer diameter × 0.25 mm inner diameter, JR‐T‐084‐M1.5, Vici Jour) was inserted 2 mm below the surface of the yeast suspension level to allow substrate injection without introducing air bubbles. When the NMR tube was positioned inside the spectrometer, 248 μL of the deuterated substrate solutions (10–30 mM, prepared in 100 mM PBS) were injected immediately through the capillary using a 1‐mL syringe. The injection was done manually within ~3 s, resulting in a visibly well‐mixed yeast sample. NMR acquisition commenced immediately after substrate injection. After each experiment, the pH of each yeast sample was measured.

All substrate concentrations reported throughout the text refer to their final values after mixing with yeast suspension. The settling of the yeast could have affected the metabolic conversion rates, which was not considered here.

The SNR of the residual water HDO peak corresponding to about 15 mM [D] (see Section 2.11) in yeast solution was about 20, measured with a single scan, indicating that concentrations below 1 mM are already challenging and accessible only with significant signal averaging that reduces time resolution.

**FIGURE 1:**
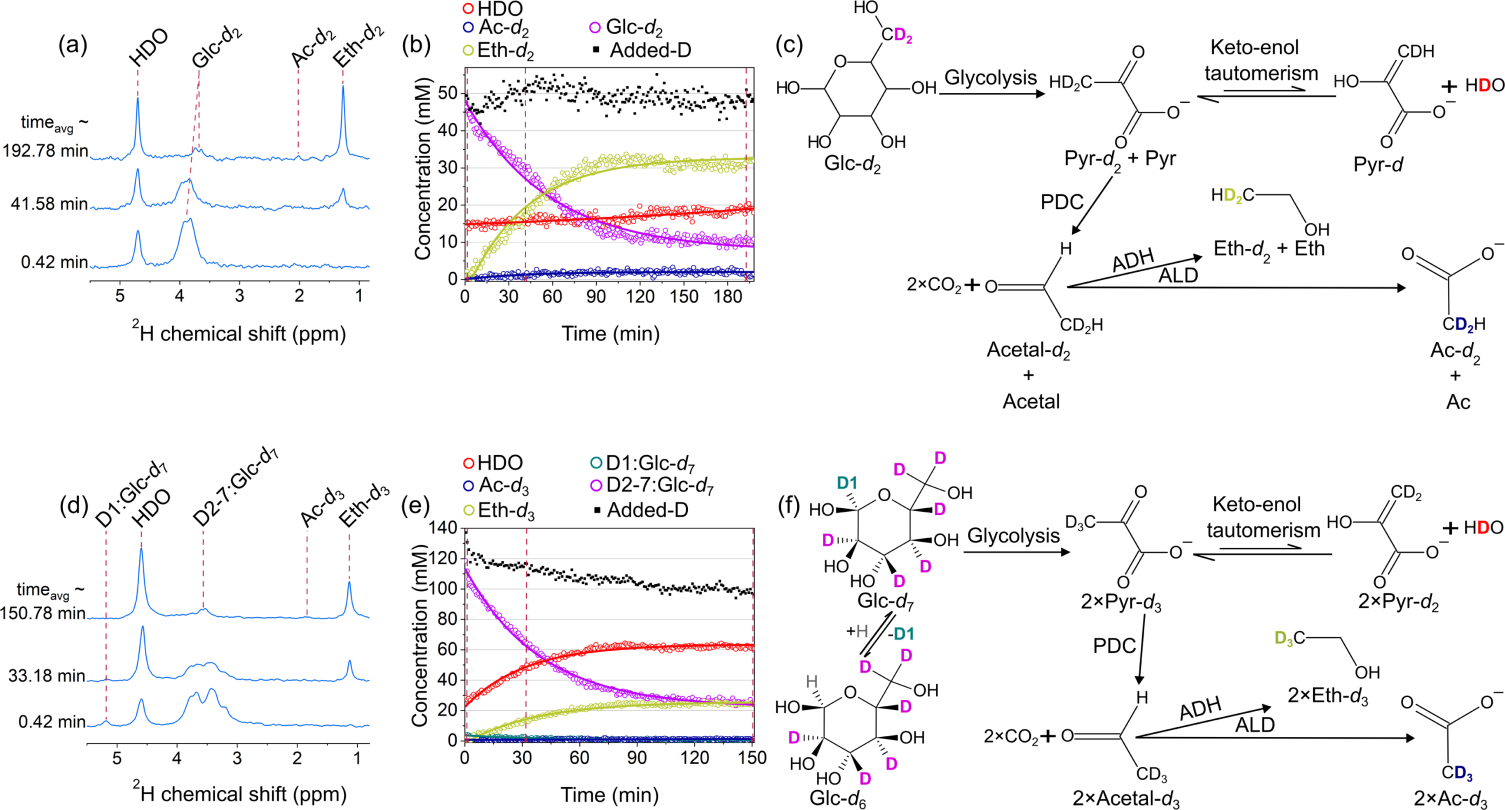
Tracking [6,6′‐^2^H_2_] and [U‐^2^H_7_]glucose (Glc*‐d*_2_ and Glc‐*d*_7_) metabolism in yeast using DMRS. ^2^H NMR spectra (a, d), the time course of metabolite signals with fitted decay functions (b, e), and schematic views of deuterium flux from Glc*‐d*_2_ (c) and Glc*‐d*_7_ (d) to [2,2‐^2^H_2_]ethanol (Eth‐*d*_2_), [2,2‐^2^H_2_] acetate (Ac‐*d*_2_) (c) and [2,2,2‐^2^H_3_]ethanol (Eth‐*d*_3_), [2,2,2‐^2^H_3_] acetate (Ac‐*d*_3_) (f). Reaction intermediates such as [3,3‐^2^H_2_]pyruvate (Pyr‐*d*_2_), [2,2‐^2^H_2_]acetaldehyde (Acetal‐*d*_2_), [3,3,3‐^2^H_3_]pyruvate (Pyr‐*d*_3_), and [2,2,2‐^2^H_3_]acetaldehyde (Acetal‐*d*_3_) were not detected, likely due to rapid metabolic conversion or low MR signal intensity. Exchange with protons resulted in a loss of deuterium label and an increase in the water peak, which is more evident in the case of Glc*‐d*_7_ (d, e). Kinetics (symbols in b and e) were obtained by integrating the corresponding lines. The concentrations were calculated using Equation (1) as described in Section 2. Added‐D value corresponds to the sum of integrals over water, ethanol, glucose, and acetate lines minus the initial water signal of 15 mM. The decrease in added‐D value is attributed to a deuterium flux to other metabolites and proteins, which are below the detection limit or too broad to quantify. The lines in (b and e) are the mono‐exponential decay fit. Enzymes involved in metabolism are pyruvate decarboxylase (PDC), alcohol dehydrogenase (ADH), and aldehyde dehydrogenase (ALD). Each spectrum was recorded over 50.4 s by averaging eight scans with 6.3 s TR for both isotopic forms. 4.7 ppm HDO resonance was used as a reference. The PBS concentration was 50 mM, and the pH levels at the experiment's beginning and end were 5.85 and 5.23, respectively (a,b, Glc*‐d*_2_) and 5.75–5.52, respectively (d,e, Glc*‐d*_7_).

**FIGURE 2:**
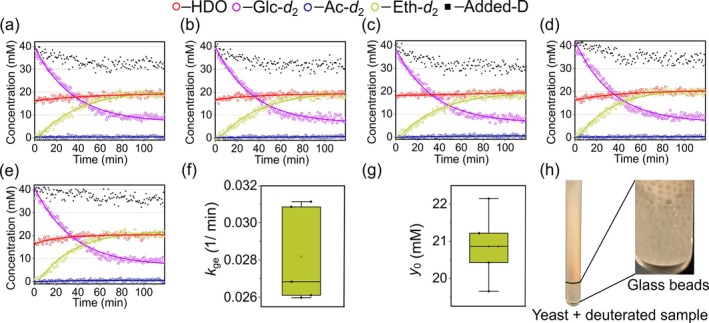
Reproducibility of DMRS with yeast in an economy NMR tube with glass beads. Kinetics of [6,6′‐^2^H_2_]glucose (Glc*‐d*_2_) metabolism with fitted decay functions (lines, Equation 3) across five independent replicates showing consistent [2,2‐^2^H_2_]ethanol (Eth‐*d*_2_) production along with minor [2,2‐^2^H_2_]acetate (Ac‐*d*_2_) formation (a–e). A small standard deviation of fitted glucose‐to‐ethanol conversion rate constants, *k*_ge_, (f), and the final Eth‐*d*_2_ concentrations, *y*_0_, (g), confirm the method's reproducibility (Table S3). The relative standard deviations for *k*_ge_ and *y*_0_ are 8.5% and 4.4%. A photo of an NMR tube containing glass beads, yeast cells, and deuterated sample solution was taken after one of these experiments (h).

**FIGURE 3:**
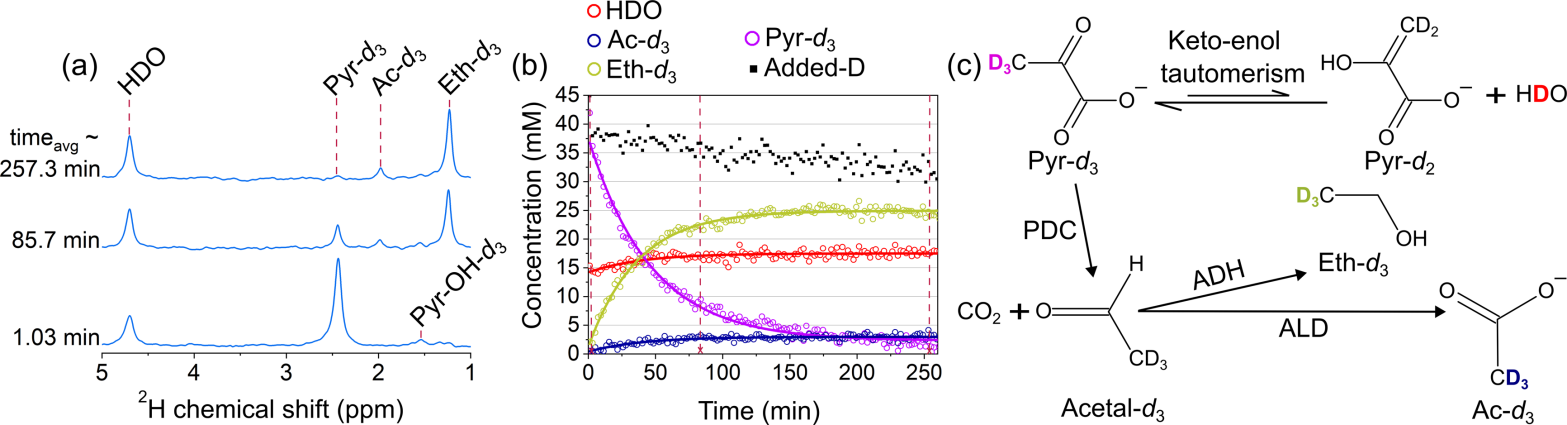
Tracking [3,3,3‐^2^H_3_]pyruvate (Pyr‐*d*_3_) metabolism in yeast using DMRS. ^2^H NMR spectra (a), the time course of metabolite signals with fitted decay functions (b), and a schematic view of deuterium flux from [3,3,3‐^2^H_3_]pyruvate (Pyr‐*d*_3_) metabolism to [2,2,2‐^2^H_3_]ethanol (Eth‐*d*_3_) and [2,2,2‐^2^H_3_]acetate (Ac‐*d*_3_) (c). Eth‐*d*_3_ and Ac‐*d*_3_ were clearly observable, whereas [2,2,2‐^2^H_3_]acetaldehyde (Acetal‐*d*_3_), an intermediate in the pathway, was not detected due to rapid metabolic conversion or low MR signal intensity. Exchange with protons resulted in a loss of deuterium label and in about a 16% increase in the water peak (a, b). Kinetics (symbols in b) were obtained by integrating the corresponding spectral lines and assigning the initial value of the water peak to 15 mM. The concentrations were calculated using Equation (1) as described in Section 2. Added‐D value corresponds to the sum of integrals over water, ethanol, pyruvate, and acetate lines minus the initial water signal of 15 mM. The decrease in added‐D value is attributed to a flux of deuterium to other metabolites and proteins, which are below the limit of detection or too broad to quantify. The lines in (b) are the mono‐exponential fit. Enzymes involved in metabolism are pyruvate decarboxylase (PDC), alcohol dehydrogenase (ADH), and aldehyde dehydrogenase (ALD). Each spectrum point was recorded every 124 s, averaging eight scans with 15.5 s TR. 4.7 ppm HDO resonance was used as a reference. The PBS concentration was 50 mM, and the pH levels at the experiment's beginning and end were 5.90 and 5.62, respectively.

**FIGURE 4:**
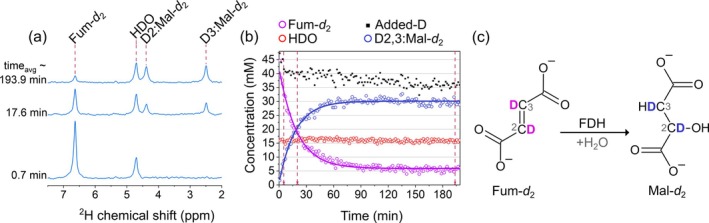
Tracking [2,3‐^2^H_2_]fumarate (Fum‐*d*_2_) metabolism in yeast using DMRS. ^2^H NMR spectra (a), the time course of metabolite signals with fitted decay functions (b), and a schematic view of deuterium flux from Fum‐*d*_2_ to [2,3‐^2^H_2_]malate (Mal‐*d*_2_) (c). There is no visible increase in a water signal (a, b). Kinetics were obtained by integrating the corresponding spectral lines (symbols in b) and assigning the initial value of the water peak to 15 mM. The concentrations were calculated using Equation (1) as described in the methods. Added‐D value corresponds to the sum of integrals over water, fumarate, and malate lines minus the initial water signal of 15 mM. The decrease in the added‐D value is attributed to a deuterium flux to other metabolites and proteins, which are below the limit of detection or too broad to quantify. The lines in (b) are the mono‐exponential decay fit with a shared exchange rate constant. Fumarase/fumarate dehydratase (FDH) enzyme drives fumarate to malate reaction. Each spectrum point was recorded every 92 s, averaging eight scans with 11.5‐s TR. 4.7 ppm HDO resonance was used as a reference. The PBS concentration was 50 mM, and the pH levels at the experiment's beginning and end were 5.90 and 5.86, respectively.

**FIGURE 5:**
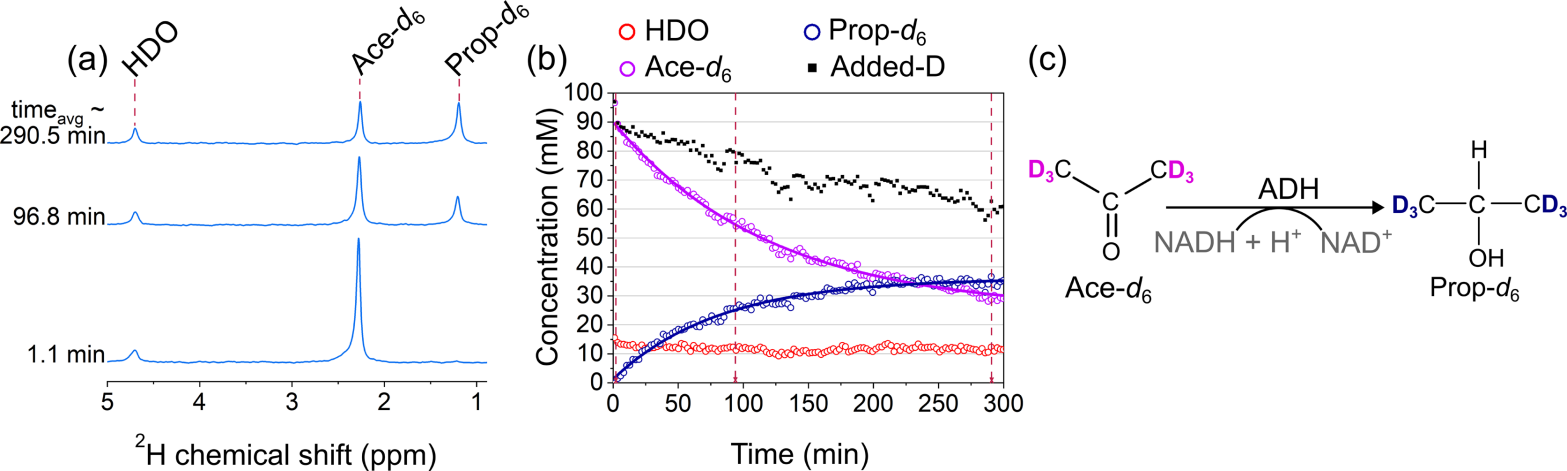
Tracking [U‐^2^H_6_]acetone (Ace‐*d*_6_) metabolism in yeast using DMRS. ^2^H NMR spectra (a), time course of metabolite signals with fitted decay functions (b), and schematic view of deuterium flux from Ace‐*d*_6_ to [1,1,1,3,3,3‐^2^H_6_]propan‐2‐ol (Prop‐*d*_6_) (c). There is no visible increase in a water signal (a, b). Kinetics were obtained by integrating the corresponding spectral lines and assigning the initial value of the water peak to 15 mM. The concentrations were calculated using Equation (1) as described in Section 2. Added‐D value corresponds to the sum of integrals over water, acetone, and propan‐2‐ol lines minus the initial water signal of 15 mM. The decrease in the added‐D value is attributed to a flux of deuterium to other metabolites and proteins that are below the limit of detection or too broad to quantify, as evaporation of acetone and propan‐2‐ol. The lines in (b) are the mono‐exponential decay fit with a shared exchange rate constant. The alcohol dehydrogenase (ADH) enzyme drives the described chemical transformation. Each spectrum point was recorded every 140 s, averaging eight scans with 17.5‐s TR. 4.7 ppm HDO resonance was used as a reference. The PBS concentration was 50 mM, and the pH levels at the experiment's beginning and end were 5.90 and 5.02, respectively.

**FIGURE 6:**
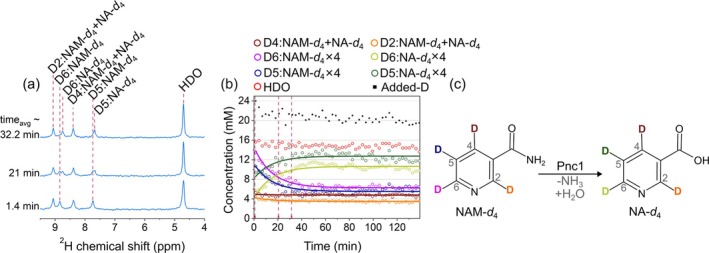
Tracking [2,4,5,6‐^2^H_4_]nicotinamide (NAM‐*d*_4_) metabolism in yeast using DMRS. ^2^H NMR spectra (a), time course of metabolite signals with fitted decay function (b), and schematic view of deuterium flux from NAM‐*d*_4_ to [2,4,5,6‐^2^H_4_]nicotinic acid (NA‐*d*_4_) (c). There is no visible increase in a water signal (a, b). Kinetics (symbols in b) were obtained by integrating the corresponding spectral lines. The concentrations were calculated using Equation (1) as described in Section 2. Added‐D value corresponds to the sum of integrals over water, NAM, and NA lines minus the initial water signal of 15 mM. The decrease of the added‐D value is attributed to a flux of deuterium to other metabolites and proteins, which are below the limit of detection or too broad to quantify. The lines in (b) are the mono‐exponential decay fit with a shared exchange rate constant. Note that NAM and NA resonances are very close: 9.094, 8.876, 8.420, and 7.772 ppm for NAM and 9.031, 8.714, 8.376, and 7.659 ppm for NA, and *D*5 and *D*6 are the most susceptible to molecular transformation. Alcohol Pnc1 enzyme drives NAM‐*d*_4_ to NA‐*d*_4_ reaction. Each spectrum point was recorded every 168 s, averaging 80 scans with 2.1‐s TR. 4.7 ppm HDO resonance was used as a reference. The PBS concentration was 50 mM, and the pH at the end and beginning of the experiment were 5.98 and 5.48, respectively.

**FIGURE 7:**
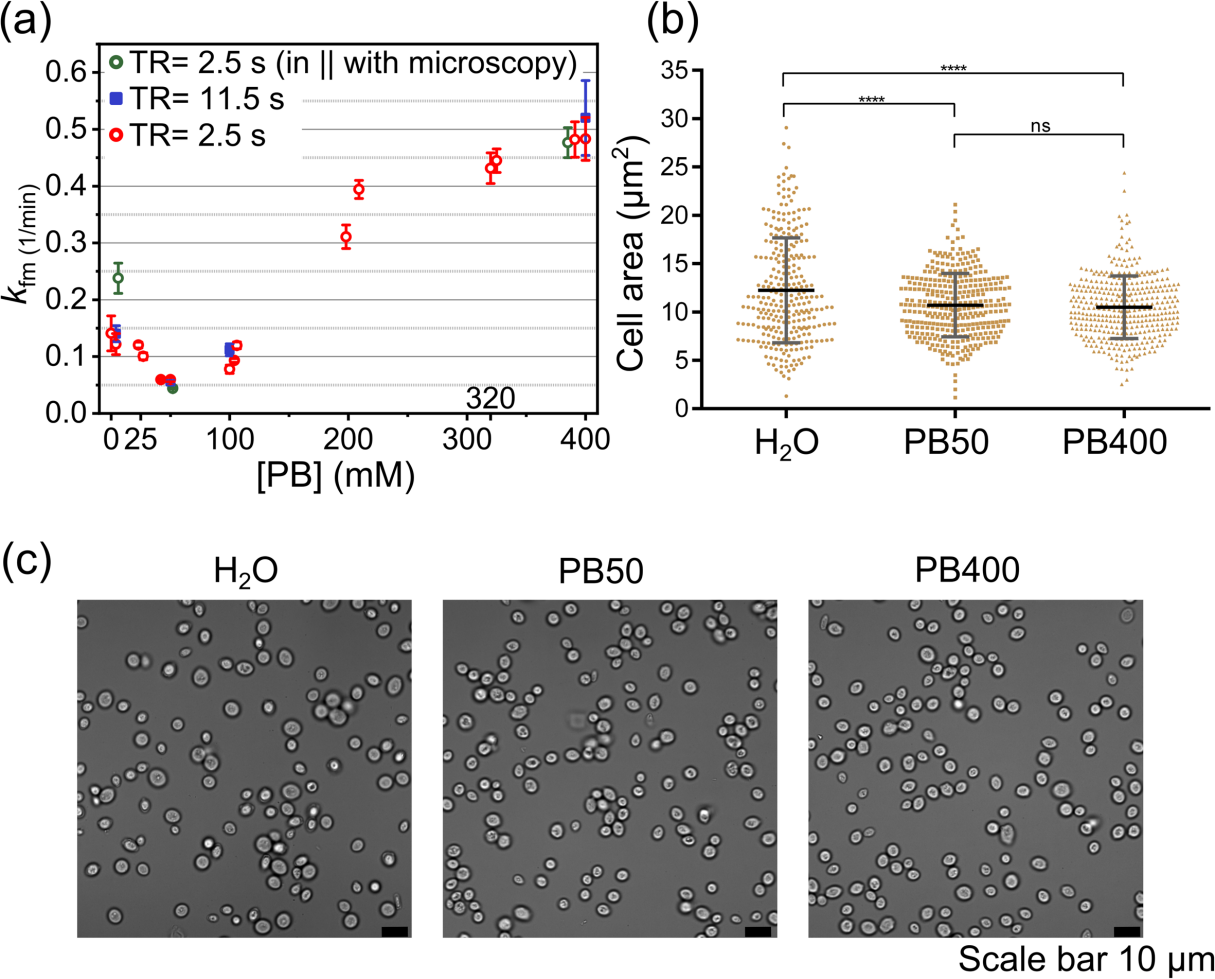
Impact of osmotic stress on yeast. Fumarate‐to‐malate conversion rate, *k*_fm_, of yeast cells (a) and microscopic study: cell area (b) and images (c) at different PBS concentrations. Seven PBS concentrations: 0, 25, 50, 100, 200, 320, and 400 mM, and three TR values of 11.5 s (blue), 2.5 s (red), and 2.5 s (green) were tested with DMRS. The slowest *k*_fm_ was observed at 50 mM [PB], whereas lower or higher [PB] accelerated *k*_fm_. No effect from TR was observed. The data points shown in green represent experiments performed in parallel with cell counting. The error margins (a) are standard errors resulting from the fitting of the fumarate to malate conversion. The symbols were moved apart from the specified [PB] values for better visualization. (b) An unpaired *t*‐test revealed a significant difference in the area of cells between H_2_O and both PB 50 mM and PB 400 mM groups (*p* < 0.0001). The size difference between the two PB groups was not statistically significant (*p* ≥ 0.05).

**FIGURE 8:**
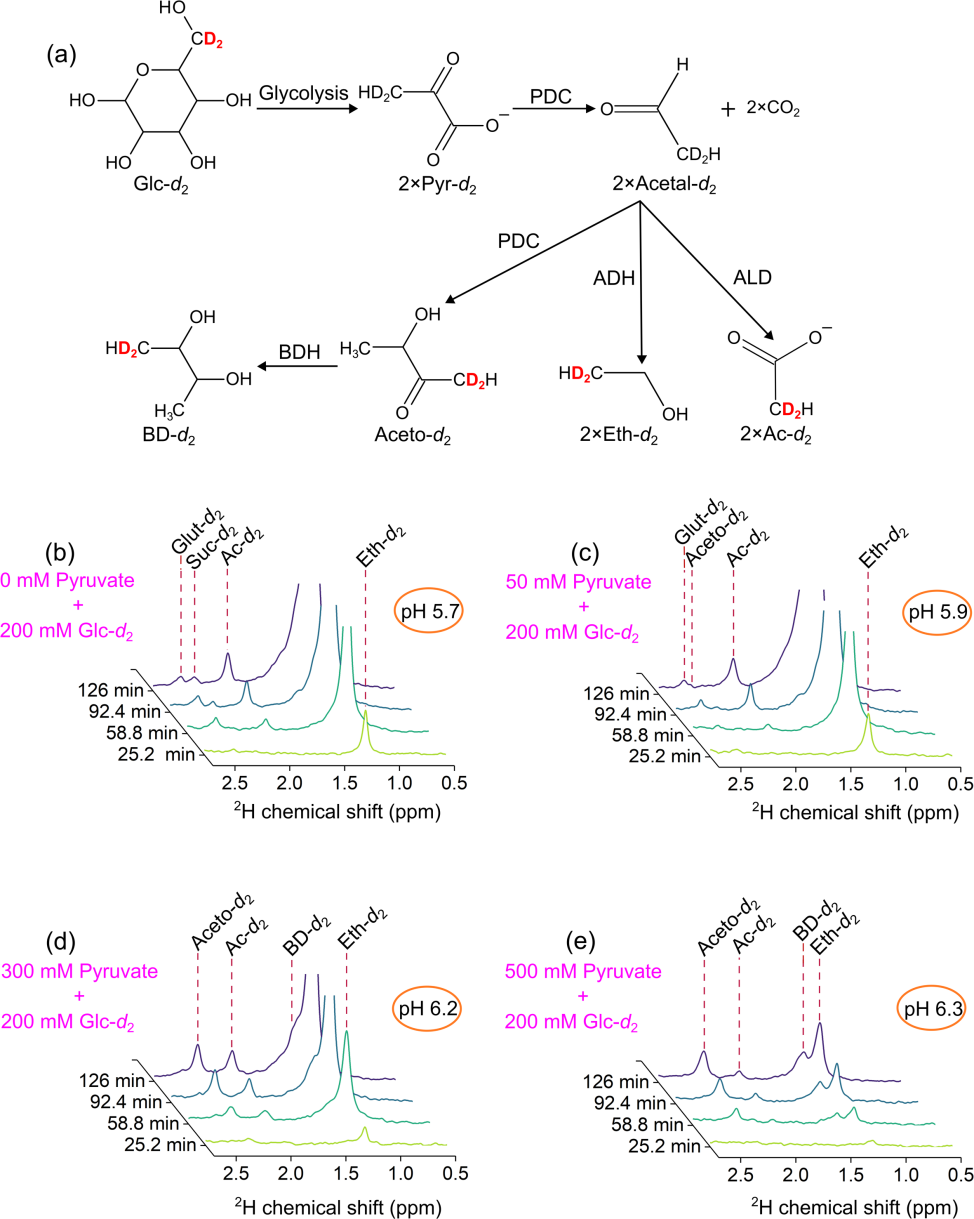
Reaction pathway (a) and DMRS kinetics of [6,6′‐^2^H_2_]glucose (Glc*‐d*_2_) metabolism in yeast assay as a function of added pyruvate to the yeast media: 0 mM (b), 50 mM (c), 300 mM (d), and 500 mM (e). The addition of pyruvate decreased the production of [2,2‐^2^H_2_]ethanol (Eth‐*d*_2_) (1.27 ppm) down to 14.2% from the initial value (from b to e), reduced the amount of [2,2‐^2^H_2_]acetate (Ac‐*d*_2_) (1.86 ppm) and [4,4‐^2^H_2_]glutamate (Glut‐*d*_2_) (2.47 ppm) /[2,3‐^2^H_2_]succinate (Suc‐*d*_2_) (2.30 ppm), and increased production of [1,1‐^2^H_2_]acetoin (Aceto‐*d*_2_) (2.32 ppm) and [1,1‐^2^H_2_]butanediol (BD‐*d*_2_) (1.42 ppm). Each spectrum was recorded every 1008 s = 16.8 min: NS = 160, TR = 6.3 s. 4.7 ppm HDO resonance was used as a reference. The PBS concentration was 50 mM. As indicated in the figure, the final pH values varied because of the added sodium pyruvate that buffered the solution. The glucose and water signals are not shown here to demonstrate the small metabolites found in this experiment better. Enzymes involved: pyruvate decarboxylase (PDC), alcohol dehydrogenase (ADH), aldehyde dehydrogenase (ALD), and butanediol dehydrogenase (BDH). *Note:* Reaction intermediates such as [3,3‐^2^H_2_]pyruvate (Pyr‐*d*_2_) and [2,2‐^2^H_2_]acetaldehyde (Acetal*‐d*_2_) were not detected, likely due to rapid metabolic conversion or low MR signal intensity.

Twenty‐two deuterated substrates, namely, Ac‐*d*_3_, Acetal‐*d*_4_, Ace‐*d*_6_, ACN‐*d*_3_, Ala‐*d*_3_, DMSO‐*d*_6_, Eth‐*d*_6_, ETX‐*d*_2_, Form‐*d*, Fum‐*d*_2_, Glc‐*d*_2_, Glc‐*d*_7_, Gly‐*d*_8_, Hyd‐Acetal‐*d*_4_, Lact‐*d*_3_, Meth‐*d*_4_, NAM‐*d*_4_, Pyr‐*d*_3_, Suc‐*d*_4_, Ser‐*d*_3_, TMSP‐*d*_4_, and Trp‐*d*_5_ (26 in total, including products and water; Table 1), were tested in yeast, and only six, Glc*‐d*_2_, Glc*‐d*_7_, Pyr‐*d*_3_, Fum‐*d*_2_, Ace‐*d*_6_, and NAM‐*d*_4_, showed visible metabolic conversion. These tracers will be discussed in Section 3, beginning with their intrinsic metabolic behavior (Figures 1, 2, 3, 4, 5, and 6), followed by selected cases where metabolism was modified by pyruvate addition (Figures 8 and S5) or osmotic stress (Figure 7).

### 2.4 Protocol for DMRS of Treated *S. cerevisiae*

This experiment followed the standard procedure with minor modifications. Yeast (0.5714 g) was suspended in 4 mL of deionized water along with varying concentrations of sodium pyruvate (P2556, Merck): 10, 50, 100, 200, 300, 400, and 500 mM, and incubated for 10 min at 32°C (~305 K), shaken every 3 min to release air. After transferring 248 μL of the suspension to a preheated NMR tube, Glc*‐d*_2_ (30, 50, and 200 mM) or NAM‐*d*_4_ (10 mM) were added. Once added, the DMRS measurement was started.

### 2.5 Preparation of PB for Fum‐*d*_2_ Osmolarity Assay

For the Fum‐*d*_2_ osmolarity assay, PB solutions were prepared at concentrations of 400, 320, 200, 100, 50, 25, and 0 mM. The 400‐mM PBS was prepared by using 280‐mM Na_2_HPO_4_ (S5011, Sigma Aldrich) and 120‐mM NaH_2_PO_4_ (S9763, Sigma Aldrich) with an adjusted pH of 7.4. Lower concentrations were prepared by serially diluting the 400‐mM PB stock with deionized water for use in various experimental conditions.

### 2.6 Microscopic Imaging

400× diluted yeast samples were placed in eight‐well chambered cover glass (Nunc Lab‐Tek Chambered Cover Glass, Catalog number: 155383, Thermo Scientific) and then imaged with the Thunder imager 3D assay (Leica Microsystems GmbH) based on a Leica DMi8 microscope with a 63× objective lens (HC PL APO 63×/1.40–0.60 OIL) and Leica K5 camera (4.2 MP, 40 FPS). The images produced were processed using Leica LAS X software.

### 2.7 Cell Counting

The yeast solution was diluted 100‐fold and mixed with 0.4% trypan blue solution in a 1:1 ratio to distinguish viable and nonviable cells, resulting in a dilution of 200. Subsequently, this mixture was loaded into a Neubauer counting chamber, and yeast cells were counted under a 20× objective lens of a Leica DM IL microscope (Leica Microsystems GmbH) within four representative small squares located at the center of the grid (16 squares total with a volume of 0.1 mm^3^). The cell concentration was consequently determined as

$$\text{Yeast cells}/1\;\mathrm{mL}=\text{Counted cells}\times \text{Dilution}\times 10^{4}$$

### 2.8 NMR Data Acquisition

The analysis of deuterium‐labeled substrates was performed using a Bruker Advance NEO (WB400) NMR spectrometer with a magnetic field strength of 9.4 T, corresponding to the ^2^H Larmor frequency of 61.4 MHz, and using Bruker Topspin 4.1.4 software. The 5‐mm broadband probe head (PA BBO 400W1/S2 BBF‐H‐D‐05 Z SP) was utilized for the experiments. We used a BB channel without a ^2^H signal trap filter to maximize the SNR of DMRS. No lock signal was used during the experiment.

Initially, the NMR parameters for the deuterium‐enriched substrates, including spin–lattice relaxation time (*T*_1_), spin–spin relaxation time (*T*_2_), were determined using inversion recovery (IR) and Carr‐Purcell‐Meiboom‐Gill (CPMG) protocols.

Relaxation parameters were determined as follows:

*T*_1_ measurements: The same IR pulse sequence was employed for all compounds with 32 pseudo‐logarithmically spaced delay times ranging from 0.01 to 25 s (0.01, 0.02, 0.04, 0.06, 0.07, 0.09, 0.1, 0.2, 0.25, 0.3, 0.35, 0.4, 0.45, 0.5, 0.6, 1.0, 1.5, 2.0 2.5, 3.0, 4.0, 5.0, 6.0, 8.0, 10, 13, 25). Each delay was acquired with two scans (NS = 2) with a pre‐excitation delay (D1) of 30 s and an acquisition time of 4 s.
*T*_2_ measurements: A standard CPMG pulse sequence was used for all compounds with 28 variable echo train lengths ranging from 4 to 9000 echoes (4, 8, 12, 16, 20, 24, 32, 40, 48, 56, 64, 72, 96, 128, 196, 256, 384, 512, 768, 1024, 1536, 2048, 3972, 4096, 5237, 6000, 7500, 9000). An echo time of 1 ms was used in each case. The duration of 180° refocusing pulse varied depending on the calibrated 90° pulse length for each experiment. Each echo train was acquired with eight scans (NS = 8), a pre‐excitation delay (D1) of 30 s, and an acquisition time of 4 s.

These measurements were carried out in a protic aqueous solution of 100 mM pH 7.4 PBS at 310 K, with substrate concentrations ranging from 10 to 30 mM. The chemical shifts (ppm) were calibrated against the residual water HDO signal, which was set to 4.7 ppm (Table 1).

In the yeast experiment, real‐time ^2^H NMR spectra and reaction tracking series of ^2^H‐labeled substrates and their metabolic products were acquired using ^2^H 90° flip angle excitation pulses, ^1^H decoupling, a receiver gain of 101, a spectral width (SW) of 133.4 ppm, and 16 k points in the time domain (TD). Each spectrum was recorded over 20–170 s by adding up 8–80 NMR transients acquired with a repetition time (TR) of 2.1–17.5 s, adjusted based on their observed *T*_1_ values of the substrates and products (Table 1). The ^2^H NMR spectra were recorded continuously for up to 350 min after injection. pH was measured at the beginning and the end of each experiment.

### 2.9 NMR Data Processing

The ^2^H NMR data were zero filled to 32 k number of points and an exponential line broadening of 3 Hz, followed by phase correction. The processed data were then analyzed using MNova software (version 14.2.2, Mestrelab Research S.L.), and selected spectra were used for data visualization.

### 2.10 Concentration of Deuterium in Water

The concentration of neat water is [H_2_O] = 1 (kg/L)/18 (g/mol) ~ 55.56 mol/L. The natural abundance of deuterium is *f*_D_ = 0.0156%. That results in the concentration of deuterium equal to [*D*_ref_ ] = [D in water] = [H_2_O] × (2 *f*_D_ (1 − *f*_D_) + *f*_D_^2^) ~ 0.000311 × [H_2_O] = 17.3 mM. This value was assumed as a reference value for the residual neat water HDO resonance peak intensity.

### 2.11 DMRS Concentration Calibration

Experiments were conducted using a PBS solution and glass beads, adhering to the previously established protocol. The experimental parameters were set to *TR* = 17.5 s and *NS* = 8, resulting in a total acquisition time of 5 h and the acquisition of 130 spectra in each case (Figure S10a–c). The signal intensity exhibited slight fluctuations with a standard deviation of approximately 3.67%. This signal, *S*_PBS_, was assigned to the [*D*_ref_] concentration of water. To calculate the concentration of any other deuterium signal, the following equation can be used:

(1) $$\left[D_{x}\right]=\left[D_{\mathrm{PBS}}\right]\cdot \frac{S_{x}}{S_{\mathrm{PBS}}}\cdot \frac{NS_{\mathrm{PBS}}}{NS_{x}}$$

where *NS* represents the number of scans in the corresponding experiment and *S*_x_ is the integral over the corresponding spectral line of species x and $\left[D_{\mathrm{PBS}}\right]=\left[D_{\mathrm{ref}}\right]$ = 17.3 mM, as calculated above.

Because of the substantial amount of yeast utilized, it was also necessary to assess the water signal in the presence of yeast and glass beads. Therefore, an additional experiment was conducted to measure the water signal in the presence of yeast, employing the same parameters as previously mentioned (Figure S11). The factor of reduction between these two experiments is given by *FR* = *S*_yeast_/*S*_PBS_ = 0.866, indicating that the total amount of water within the sensitive area of the coil in the presence of yeast is reduced by this factor *FR* and is equal to 17.3 mM × *R* = 15 mM.

Therefore, a more precise conversion for the concentration of compounds in the presence of yeast is given by

(2) $$\left[D_{x}\right]=\left[D_{\text{yeast}}\right]\cdot \frac{S_{x}}{S_{\text{yeast}}}\cdot \frac{{\mathrm{NS}}_{\text{yeast}}}{NS_{x}}$$

However, by substituting $\left[D_{\text{yeast}}\right]=\mathrm{FR}\left[D_{\mathrm{PBS}}\right]$ and $S_{\text{yeast}}=\mathrm{FR}\;S_{\mathrm{PBS}}$, it can be shown that this equation is equivalent to the previous conversion equation (Equation 1), meaning that the conversion equations are identical. Therefore, either equation can be used, as the observation volume remains consistent in both cases, and the volume was not considered in the calculations. Equation (1) was used in the manuscript.

### 2.12 DMRS Kinetics Fitting

The processed NMR spectra were subjected to integration using MNova software. The resulting integrals were subsequently analyzed to determine the kinetic parameters of the reaction. The integrals were fitted using OriginPro software (version 2021b, OriginLab), applying a mono‐exponential decay function:

(3) $$I\left(t\right)=A\cdot \exp\left(-t\cdot k\right)+y_{0}$$

where $I\left(t\right)$ is the integral over the spectral line at time t, A is the amplitude (difference between the initial and final values), $A+y_{0}$ is the initial integral of the signal (i.e., the concentration at time zero), k is the first‐order reaction constant, and $y_{0}$ is an asymptotic (stationary) value (i.e., the concentration at infinity). For deuterated substrates, $y_{0}$ represents the residual concentration that remains unconverted. For metabolic products, $y_{0}$ represents the final concentration of the metabolite. The fitting process was based on the assumption that the reaction adheres to first‐order kinetics.

Note that for substrates, *A* > 0 represents exponential decay (disappearance of the substrate); for metabolic products, *A* < 0 represents exponential growth (appearance of the product). In both cases, the signal approaches the asymptotic value $y_{0}$, consistent with first‐order kinetics.

Using OriginPro software, standard errors (SE) of fitted parameters were acquired from the covariance matrix of the nonlinear least squares fit; in the case of fitting, SE coincides with standard deviation (SD).

In the case of NAM‐*d*_4_, Fum‐*d*_2_, and Ace‐*d*_6_ conversions, when only a single product was observed, the fitting with a shared conversion rate constant for the reagent and product was used.

## 3 Result and Discussion

### 3.1 ^2^H NMR Parameters of Selected Tracers

Deuterium chemical shifts, *T*_1_ and *T*_2_ relaxation constants of 25 compounds in PBS were determined at 9.4 T and 310 K (Table 1 and spectra in Figure S9a–x). The list includes Ac‐*d*_3_, Acetal‐*d*_4_, Ace‐*d*_6_, ACN‐*d*_3_, Ala‐*d*_3_, DMSO‐*d*_6_, Eth‐*d*_6_, ETX‐*d*_2_, Form‐*d*, Fum‐*d*_2_, Glc‐*d*_2_, Glc‐*d*_7_, Gly‐*d*_8_, Hyd‐Acetal‐*d*_4_, Lact‐*d*_3_, Meth‐*d*_4_, NAM‐*d*_4_, Pyr‐*d*_3_, Ser‐*d*_3_, Suc‐*d*_4_, TMSP‐*d*_4_, Trp‐*d*_5_, yeast's metabolic products, [2,3‐^2^H_2_]malate (Mal‐*d*_2_), [2,4,5,6‐^2^H_4_]nicotinic acid (NA‐*d*_4_), [1,1,1,3,3,3‐^2^H_6_]propan‐2‐ol (Prop‐*d*_6_), and water, totaling 26 molecules.

**TABLE 1:** ^2^H NMR parameters (*T*_1_, *T*_2_, and chemical shift) for 26 biomolecules, drugs, metabolites, and solvents at 9.4 T and 310 K.

#	Tracers	Abbreviation	Chemical shift, ppm	*T*_1_, ms	*T*_2_, ms
1	[2,2,2‐^2^H_3_]acetate	Ac‐*d*_3_	1.867 [CD_3_]	3140 ± 40 [CD]	3300 ± 300 [CD_3_]
2	[1,2,2,2‐^2^H_4_]acetaldehyde	Acetal‐*d*_4_	9.849 [CD], 2.353 [CD_3_]	3675 ± 374 [CD], 4392 ± 235 [CD_3_], 4033 ± 304 [tot]	3310 ± 226 [CD(1)], 4761 ± 131 [CD_3_(2)], 4035 ± 178 [tot]
3	[U‐^2^H_6_]acetone	Ace‐*d*_6_	2.323 [CD_3_]	3532 ± 20 [CD_3_]	3487 ± 248 [CD_3_]
4	[2,2,2‐^2^H_3_]acetonitrile	ACN‐*d*_3_	2.173 [CD_3_]	5322 ± 84 [CD_3_]	5433 ± 163 [CD_3_]
5	[3,3,3‐^2^H_3_]alanine	Ala‐*d*_3_	1.566 [CD_3_]	308 ± 5 [CD_3_]	282 ± 3431 [CD_3_]
6	[U‐^2^H_6_]dimethyl sulfoxide	DMSO‐*d*_6_	2.684 [CD_3_]	930 ± 20 [CD_3_]	970 ± 30 [CD_3_]
7	[U‐^2^H_6_]ethanol	Eth‐*d*_6_	3.605[CD_2_(1)], 1.115 [CD_3_(2)]	1650 ± 3 [CD_2_(1)], 1310 ± 20 [CD_3_(2)]	1400 ± 100 [CD_2_(1)], 1250 ± 60 [CD_3_(2)]
8	[4,4′‐^2^H_2_]ethosuximide	ETX‐*d*_2_	2.726 [CD(4)], 2.573 [CD(4′)]	270 ± 10 [CD(4)], 300 ± 20 [CD(4′)], 290 ± 20 [tot]	300 ± 30 [CD(4)], 270 ± 30 [CD(4′)], 270 ± 20 [tot]
9	[1‐^2^H]formate	Form‐*d*	8.46 [CD]	1880 ± 60 [CD]	1700 ± 160 [CD]
10	[2,3‐^2^H_2_]fumaric acid	Fum‐*d*_2_	6.653 [CD(2, 3)]	247 ± 2 [CD(2, 3)]	246 ± 18 [CD(2, 3)]
11	[6,6′‐^2^H_2_]glucose	Glc‐*d*_2_	3.844 [CD(6)], 3.975 [CD(6′)]	77 ± 2 [tot]	77 ± 5 [tot]
12	[U‐^2^H_7_]glucose	Glc‐*d*_7_	5.275 [CD(1), α‐anomer] 4.75 [CD(1), β‐anomer]—water overlap 3.36–3.92 [CD(2,3,4,5,6,6′)]—unresolved	73 ± 5 [CD(1), α‐anomer], 75 ± 3 [tot of CD(2,3,4,5,6,6′)]	55 ± 10 [CD(1), α‐anomer], 63 ± 6 [tot of CD(2,3,4,5,6,6′)]
13	[U‐^2^H_8_]glycerol	Gly‐*d*_8_	3.893 [CD(2)], 3.75–3.66 [CD_2_(1, 3)]—unresolved	267 ± 2 [tot]	255 ± 6 [tot]
14	[1,2,2,2‐^2^H_4_]hydrated‐acetaldehyde	Hyd‐Acetal‐*d*_4_	5.377 [CD], 1.430 [CD_3_]	374 ± 26 [CD], 426 ± 10 [CD_3_], 400 ± 18 [tot]	394 ± 216 [CD], 427 ± 1 33 [CD_3_], 410 ± 174 [tot]
15	[3,3,3‐^2^H_3_]lactate	Lact‐*d*_3_	1.397 [CD_3_]	341 ± 3 [CD_3_]	340 ± 102 [CD_3_]
16*	[2,3‐^2^H_2_]malate (pH 5.86)	Mal‐*d*_2_	4.368 [CD(2)], 2.474 [CD(3)]	153 ± 9 [CD(2)], 142 ± 10 [CD(3)], 147 ± 10 [tot]	152 ± 23 [CD(2)], 140 ± 25 [CD(3)], 146 ± 35 [tot]
17	[U‐^2^H_4_]methanol	Meth‐*d*_4_	3.309 [CD_3_]	4600 ± 200 [CD_3_]	4200 ± 60 [CD_3_]
18	[2,4,5,6‐^2^H_4_]nicotinamide	NAM‐*d*_4_	9.094 [CD(2)], 8.876 [CD(6)], 8.420 [CD(4)], 7.772 [CD(5)]	199 ± 13 [CD(2)], 147 ± 10 [CD(6)], 208 ± 17 [CD(4)], 173 ± 10, [CD(5)], 182 ± 12 [tot]	180 ± 68 [CD(2)], 66 ± 14 [CD(6)], 153 ± 54 [CD(4)], 262 ± 102, [CD(5)], 165 ± 59 [tot]
19*	[2,4,5,6‐^2^H_4_]nicotinic acid (pH 5.48)	NA‐*d*_4_	9.031 [CD(2)], 8.714 [CD(6)], 8.376 [CD(4)], 7.659 [CD(5)]	201 ± 40 [CD(2)], 146 ± 22 [CD(6)], 225 ± 37 [CD(4)], 230 ± 32 [CD(5)], 200 ± 32 [tot]	177 ± 85 [CD(2)], 59 ± 25 [CD(6)], 147 ± 83 [CD(4)], 228 ± 47 [CD(5)], 152 ± 63 [tot]
20*	[1,1,1,3,3,3‐^2^H_6_]propan‐2‐ol (pH 5.02)	Prop‐*d*_6_	1.201 [CD_3_]	713 ± 25 [CD_3_]	711 ± 80 [CD_3_]
21	[3,3,3‐^2^H_3_]pyruvate	Pyr‐*d*_3_	2.468 [CD_3_]	2010 ± 30 [CD_3_]	1950 ± 115 [CD_3_]
22	[2,3,3′‐^2^H_3_]serine	Ser‐*d*_3_	3.805 [CD(2)], 3.949 [CD(3)], 3.902 [CD(3′)]	199 ± 3 [CD(2)], 183 ± 2 [CD(3,3′)], 188 ± 2 [tot]	196 ± 5 [CD(2)], 173 ± 3 [CD(3,3′)], 183 ± 3 [tot]
23	[2,2,3,3‐^2^H_4_]succinic acid	Suc‐*d*_4_	2.474 [CD_2_(2, 3)]	288 ± 2 [CD_2_(2, 3)]	285 ± 13 [CD_2_(2, 3)]
24	[2,2,3,3‐^2^H_4_]trimethylsilylpropionic acid	TMSP‐*d*_4_	2.078 [CD_2_(2)], 0.699 [CD_2_(3)]	300 ± 10 [CD_2_(2)], 300 ± 10 [CD_2_(3)]	290 ± 20 [CD_2_(2)], 260 ± 20 [CD_2_(3)]
25	[2,4,5,6,7‐^2^H_5_]tryptophan	Trp‐*d*_5_	7.8827 [CD(4)], 7.7250 [CD(7)] 7.37–7.47 [CD(2, 5, 6)]—unresolved	58 ± 0.827 [tot]	65 ± 0.969 [tot]
26	[^2^H]water	HDO	4.70	630 ± 20	610 ± 50

*Note:* The wide range of *T*_1_ and *T*_2_, spanning from 70 to 4000 ms. The molecule concentrations were in the range of 10–30 mM. The chemical shifts were calibrated to the residual water resonance HDO, which was set to 4.70 ppm. When the spectra were unresolved or contained multiple lines close to each other, the total effective relaxation time for these deuterons was also estimated, denoted as “tot.” Exemplary spectra (Figure S9a–x) and a similar table for 293 K (Table S1) are available in the Supporting Information. Compounds are ordered alphabetically. Parameters were determined in 100‐mM PBS, pH 7.4, or as metabolic products of yeast marked with an asterisk (*). These molecules emerged as metabolites, not substrates, and were measured once the yeast reaction had finished: (1) [2,3‐^2^H_2_]malate (Mal*‐d*_2_) at pH 5.86 was measured as a metabolic product of [2,3‐^2^H_2_]fumarate (Fum*‐d*_2_), (2) [2,4,5,6‐^2^H_4_]nicotinic acid (NA‐*d*_4_) at pH 5.48 as a product of [2,4,5,6‐^2^H_4_]nicotinamide (NAM‐*d*_4_), and (3) [1,1,1,3,3,3‐^2^H_6_]propan‐2‐ol (Prop‐*d*_6_) at pH 5.02 as a product of [U‐^2^H_6_]acetone (Ace‐*d*_6_). These metabolites' parameters reflect slightly different conditions from other experiments.

It is often assumed that the relaxation time of deuterium is generally rapid and of the order of 100 ms. This is the case for glucose, lactate, fumarate, nicotinamide, tryptophan, and many other molecules. However, for several compounds like pyruvate, methanol, acetone, and acetaldehyde, the relaxation time exceeds 3 s, with the longest measured being 5.3 s for acetonitrile. Therefore, special care must be taken when measuring these molecules as a tracer or a product, as an inappropriate repetition time (TR, time between two consecutive excitations) may lead to false concentration estimates due to signal saturation.

For example, in our yeast experiments discussed below, *T*_1_(Glc‐*d*_2_) = (77 ± 2) ms and *T*_1_(Glc‐*d*_7_) = (75 ± 3) ms were much shorter than *T*_1_ of their metabolic product, [2,2‐^2^H_2_]ethanol (Eth‐*d*_2_), *T*_1_(Eth‐*d*_2_) = (1100 ± 5) ms. In the case of Pyr‐*d*_3_, the observed *T*_1_(Pyr‐*d*_3_) = (2010 ± 30) ms, in between its metabolic products: *T*_1_(Ac‐*d*_3_) = (3140 ± 4) ms and *T*_1_ of [2,2,2‐^2^H_3_]ethanol (Eth‐*d*_3_) was (1102 ± 5) ms. Note that in vivo *T*_1_ of Glc*‐d*_2_ is even shorter [47, 60]; hence, the values measured here in the PBS solution (Table 1) correspond to an upper boundary.

These findings are essential to plan and interpret DMRS experiments using a sufficiently long TR of NMR acquisition to allow for direct quantitative DMRS without signal saturation.

### 3.2 Glucose Metabolism in Yeast

Glucose is the fundamental component in cellular energy metabolism and has been exploited widely as a metabolic probe in several diseased conditions [47, 48, 85, 86, 87]. Therefore, it was our first tested substrate.

We observed extended real‐time metabolism of Glc*‐d*_2_ and Glc*‐d*_7_. The deuterium label was traced across several metabolic steps and detected in fermentative catabolic products, mainly Eth‐*d*_2_ and [2,2‐^2^H_2_] acetate (Ac‐*d*_2_) for Glc*‐d*_2_ (Figure 1a,b,c) and Eth‐*d*_3_, Ac‐*d*_3_ isotopomers for Glc‐*d*_7_ (Figure 1d,e,f).

The first ^2^H NMR spectrum, immediately after the Glc*‐d*_2_ and Glc*‐d*_7_ incorporation, displayed poorly resolved deuterium resonances of Glc*‐d*_2_ at 3.8 ppm and Glc*‐d*_7_ at 3.771, 3.510, 5.275 ppm (Figure 1a,d). In subsequent spectra, the main products Eth‐*d*_2_ and Eth‐*d*_3_ appeared with the resonance at about 1.25 ppm. A much smaller resonance appeared at about 2.0 ppm, corresponding to Ac‐*d*_2_ and Ac‐*d*_3_. No other resonances were observed in these spectra apart from the natural abundance HDO resonance at 4.7 ppm, which was considered the internal standard during the experiment.

Although DMRS appears to be quantitative, due to the rapid deuterium–proton chemical exchange in some cases, it can be challenging to measure the exact concentration of the molecule. Therefore, in all kinetics presented below (e.g., Figure 1b,e), we report the deuterium concentration, which in some cases can be converted into concentrations of molecules by dividing by the corresponding number of deuterons in the molecule.

#### 3.2.1 Quantification of Ethanol Production From Glucose by Yeast

When 24 mM of Glc*‐d*_2_ was present in the solution, [HDO] increased from the baseline 15 mM value to 18 mM in 3 h (Figure 1b). In contrast, [HDO] rose rapidly to 62 mM when 19 mM of Glc*‐d*_7_ was present (Figure 1e). The difference in [HDO] production between Glc*‐d*_7_ and Glc*‐d*_2_ is attributed to their distinct metabolic pathways and the positioning of deuterium‐labeled atoms. Glc*‐d*_7_ metabolism rapidly increased [HDO] through early and downstream enzymatic reactions. This includes deuterium loss from C3 and C4 positions during the glycolytic transformation of glyceraldehyde‐3‐phosphate to 1,3‐biphosphoglycerate, and from the C1 position through the pentose phosphate pathway. In contrast, [HDO] was not affected by Glc*‐d*_2_ until downstream metabolism, such as alanine transaminase activity and TCA cycle oxidation. As a result, Glc*‐d*_7_ yielded 3.5 times more HDO per glucose molecule compared with Glc*‐d*_2_. Additionally, keto‐enol tautomerization in pyruvate at the C3 position also contributed to deuterium label loss in both cases [60].

The Eth‐*d*_2_ signal in the case of Glc*‐d*_2_ after about 3 h reached 30 mM of [D] that corresponds to at least [ethanol] = 2·½·(18/15) 30 mM = 36 mM, where the first factor “2” describes that only every second ethanol molecule produced from Glc*‐d*_2_ has deuterons, “½” because there are two deuterons per Eth‐*d*_2_, and the factor 18/15 assessed from water signal increase partially compensates for deuterium loss via exchange with water.

The “added‐D” value, defined as the sum of concentrations of all observed molecules: HDO, Glc, Ac, and Eth, minus naturally occurring 15 mM of HDO, is relatively stable and close to the initial [D] of added Glc*‐d*_2_. This indicates that deuterons of Glc*‐d*_2_ present mostly in these four observed compounds.

In the case of Glc*‐d*_7_, the situation is much more complex as the observed added‐D value decays from the initial [D] of added Glc*‐d*_7_ due to rapid deuterium label loss during the initial and subsequent steps of glycolysis and the TCA cycle, as well as its incorporation into undetectable with DMRS molecules, limiting the precision of the elucidation of chemical transformation.

A mono‐exponential decay fit was applied to the observed ethanol signals (Figure 1b,e) to obtain the production rate characteristic time of its production from glucose by yeast, yielding (0.0243 ± 0.0008) min^−1^ and (0.0271 ± 0.0008) min^−1^ in the case of Glc*‐d*_2_ and Glc*‐d*_7_, respectively. The standard deviation here is the result of fitting; experiments were performed once. Hence, no statistical comparison was made. Despite the rapid deuteron loss, the apparent ethanol build‐up rates were comparable but not identical, with a minor difference (around 11%) likely attributable to earlier deuterium shedding in Glc‐*d*_7_ metabolism.

#### 3.2.2 DMRS Reproducibility

To assess the reproducibility of the entire protocol, we repeated experiments five times with different batches of purchased commercial yeast of the same brand (Figure 2). In all cases, an identical Glc*‐d*_2_ concentration (20 mM) was administered to the NMR assembly. Each time, a new 5‐mm NMR tube was used with added glass beads.

Glc*‐d*_2_ metabolic conversion in yeast was tracked with Eth‐*d*_2_ (1.25 ppm) as the main metabolic product, and Ac‐*d*_2_ (2 ppm) as a secondary product. The HDO (4.7 ppm) resonance was considered an internal standard, and pH readings were recorded following each trial. Each spectrum point for Glc*‐d*_2_ was recorded using the same NMR acquisition parameters as those in Figure 1.

All five replicates exhibited similar Glc‐*d*_2_ conversion kinetics, as shown in Figure 2a–e. Quantitative fitting yielded a glucose‐to‐ethanol conversion rate (*k*_ge_), with a mean of 0.0282 min^−1^, a standard deviation (SD) of 0.0024, and a coefficient of variation (CV) of 8.5% (Figure 2f). Similarly, the estimated final ethanol concentration (*y*_0_) was (20.86 ± 0.91) mM (mean ± SD) with a CV of 4.4% (Figure 2g). The data demonstrate slight variability in the experimental procedure, indicating sufficiently high reproducibility (CV < 10%) of the DMRS method and the used commercial food‐grade yeast. Individual replicate data, including curve‐fitting uncertainties, are provided in Table S3.

### 3.3 Pyruvate Metabolism in Yeast

Next, we investigated the metabolic conversion of Pyr‐*d*_3_, a key intermediate between glycolysis and the TCA cycle [88]. Pyruvate decarboxylase (PDC) plays a crucial role in this process by catalyzing the decarboxylation of pyruvate in a magnesium‐dependent reaction to produce CO_2_ and the reactive intermediate acetaldehyde. Acetaldehyde is then reduced to ethanol and acetate.

PDC is a thiamine diphosphate (ThDP)–dependent enzyme that requires both ThDP and a divalent cation (Ca^2+^, Mg^2+^, or Mn^2+^) as essential cofactors. These cofactors facilitate the non‐oxidative decarboxylation of alpha‐keto acids, leading to the formation of aldehydes and hydroxyl ketones through carboligation reactions [89, 90, 91].

Following the addition of 15 mM Pyr‐*d*_3_ (2.468 ppm), the ^2^H spectra revealed the production of its major metabolites Eth‐*d*_3_ (1.2261 ppm) and Ac‐*d*_3_ (1.9775 ppm) (Figure 3a,c). As before, the HDO (4.7 ppm) signal was slowly rising due to deuteron–proton chemical exchange.

In about 200 min, yeasts consumed 13 mM out of 15 mM Pyr‐*d*_3_. The small portion of [D] ~ 3 mM contributed to the rise of the HDO signal. The Eth‐*d*_3_ signal corresponds to about 26 mM [D] or at least 26 mM/3 ~ 8.6 mM of [Ethanol] produced from pyruvate (Figure 3b). At the same time, Ac‐*d*_3_ resonance reached only about 3.1 mM [D], corresponding to at least 1‐mM acetate concentration, demonstrating 8.6/1 ~ 8.6 times higher ethanol production than acetate. Although the final concentration of acetate was smaller, the characteristic kinetic rates obtained by fitting the corresponding kinetics were similar: (0.025 ± 0.0033) min^−1^ for Ac‐*d*_3_ and (0.0274 ± 0.00065) min^−1^ for Eth‐*d*_3_. The values are similar to the ones measured with Glc*‐d*_2/7_.

### 3.4 Fumarate Metabolism in Yeast

Recently, fumarate has gained attention for its role as a sensitive tracer for imaging cell necrosis [92] and as a diagnostic tool for assessing therapeutic response [16, 66]. The reason for this is that in healthy cells, fumarate metabolism is undetectable because the transport of exogenous fumarate to the intracellular enzyme fumarase occurs too slowly for the time frame of hyperpolarized MRI. However, in necrotic cells, the membrane is permeable, giving access to intracellular fumarase, both inside and outside the cell. As a result, fumarate is efficiently converted to malate, leading to a substantial increase in malate production [66].

Upon the addition of 22 mM of Fum‐*d*_2_ (6.653 ppm), two resonance signals corresponding to ^2^H associated with C2 (2.474 ppm) and C3 (4.368 ppm) of Mal‐*d*_2_ appeared (Figure 4a,c). Unlike Glc*‐d*_2/7_ and Pyr‐*d*_3_, upon adding Fum‐*d*_2_, the intensity of HDO resonance remained stable throughout the experiment of 3 h. However, the added‐D value corresponding to the sum of integrals over water, fumarate, and malate, minus the initial water concentration of 15 mM, decreased by about 20%, indicating the production of other molecules, which were not observable.

The starting [D] of Fum‐*d*_2_ was 44 mM, which led to 31 mM of [D] of Mal‐*d*_2_ after 120 min, with the remaining [D] of Fum‐*d*_2_ being only 6.5 mM (Figure 4b). The missing 6.5 mM likely contributed to other metabolites with much smaller signal intensities. Malate was further oxidized to oxaloacetate through the malate dehydrogenase (MDH) reaction. Subsequently, oxaloacetate did not accumulate and was rapidly utilized in other metabolic pathways through the TCA cycle and gluconeogenesis. The Fum‐*d*_2_ and Mal‐*d*_2_ integrals were fitted together using mono‐exponential decay function with the shared conversion rate constant *k*_fm_ = (0.0561 ± 0.0018) min^−1^.

### 3.5 Acetone Metabolism in Yeast

Previously, acetone has been investigated in fermentation mashes by using a yeast model as a metabolic precursor molecule for producing propan‐2‐ol, a secondary alcohol catalyzed by ADH [93]. Using DMRS, we observed Prop‐*d*_6_ (1.201 ppm) real‐time production as a main metabolic product from Ace‐*d*_6_ (2.323 ppm; Figure 5a,c). HDO signal was stable during the 5 h course of the experiment, while starting with 16 mM of acetone, yeast produced about 6 mM propan‐2‐ol with the characteristic conversion rate *k*_ap_ = (0.0123 ± 0.0004) min^−1^, leaving only about 4.8 mM acetone (Figure 5b). The conversion rate is about half of the conversion rate for ethanol production from pyruvate or glucose. The Ace‐*d*_6_ and Prop‐*d*_6_ integrals were fitted together using mono‐exponential decay functions with this shared conversion rate constant.

As the added‐D value (the sum of acetone, HDO, and propan‐2‐ol) goes down, it indicates additional sources of deuterium loss. The possible reasons include the evaporation of volatile tracer (acetone) and product (propan‐2‐ol), and the production of other compounds with much smaller signal intensities.

Yeast ADH exhibits higher specificity for smaller substrates such as ethanol and acetaldehyde, where its activity is much more efficient. Although it can also catalyze the oxidation of other primary and secondary alcohols and the reduction of higher aldehydes or methyl ketones, these reactions occur to a lesser extent [94]. This lower activity is likely due to the smaller substrate‐binding pocket [95], which is less suitable for accommodating longer chain alcohols or bulkier molecules like acetone, resulting in incomplete metabolism during fermentation.

### 3.6 Nicotinamide Metabolism in Yeast

Nicotinamide (NAM) is an amide derivative of nicotinic acid (NA, niacin, vitamin B_3_). It serves as a precursor for the production of nicotinamide adenine dinucleotide (NAD) [96], a ubiquitous co‐enzyme that regulates redox states and energy levels in various cellular processes, such as the TCA cycle, glycolysis, and oxidative phosphorylation. Additionally, NAD can be consumed as a co‐substrate in DNA repair mechanisms using poly‐ADP‐ribose polymerase (PARP) [97]. Owing to its clinical safety, NAM can be utilized for in vivo investigations of the enzymatic processes involved in physiological functions and diagnostic purposes [9, 98].

In our study, upon introducing 12 mM NAM‐*d*_4_ with resulting PBS of 50 mM and pH of 5.98, the initial ^2^H resonances of NAM‐*d*_4_ appeared at 9.094, 8.876, 8.420, and 7.772 ppm. Subsequent spectra revealed the ^2^H resonances of NA‐*d*_4_, a sole observable metabolite with resonances at 9.031, 8.714, 8.376, and 7.659 ppm (Figure 6a,c, pH 5.48 at the end of the measurement). The concentration of HDO remained stable throughout the experiment.

Mono‐exponential decay fit was applied to integrals over *D*_5_ and *D*_6_ resonances of NAM and NA (Figure 6b) with shared conversion rate constant *k*_NA_ = (0.0669 ± 0.0043) min^−1^. The conversion rate constant for NAM to NA was about 2.5 times faster than for ethanol production from pyruvate or glucose.

The NAM to NA and ammonia reaction is catalyzed by nicotinamide deamidase (Pnc1), an enzyme found in yeast [98, 99]. In contrast, human cells lack Pnc1 and instead convert NAM to nicotinamide mononucleotide (NMN) via nicotinamide phosphoribosyl transferase (Nampt), an enzyme not present in yeast. However, the gut microbiota in humans can assist in converting NAM to NA through microbial deamidases [100], similar to the results observed here in yeast.

### 3.7 Effect of PB Concentration on the Metabolism of Fumarate

We set out to test the impact of osmotic stress, modeled by changing PB concentration, on the fumarate‐to‐malate conversion rate, *k*_fm_, as an indicator for cell viability. We prepared stock solutions with 0, 25, 50, 100, 200, 320, and 400 mM PB and used them to prepare the yeast assay and 30‐mM Fum‐*d*_2_ solution. Each measured metabolite signal was fitted (similar to Figure 4b), yielding *k*_fm_ as a function of [PB] (Figure 7a).

To see if the TR had an effect on the measured *k*_fm_ values, we first used a long TR of 11.5 s, which is in line with our standard Fum‐*d*_2_ experiments (Figure 4). This long TR ensured the full magnetization recovery of all Fum‐*d*_2_ and Mal‐*d*_2_; no additional resonances beyond fumarate and malate were detected. We then lowered the TR to 2.5 s to enable faster acquisitions across multiple PB concentrations. The reduced TR was still long enough for the full magnetization recovery for Fum‐*d*_2_, Mal‐*d*_2_, and HDO, while allowing more rapid signal acquisition. The unpaired *t*‐tests at matched PB concentrations (0, 50, and 400 mM) confirmed no statistically significant differences in *k*_fm_ between different TR settings (*p* = 0.95 for TR = 11.5 s vs. 2.5 s), *p*‐value > 0.05, indicating that shorter TR can be reliably used for faster data acquisition without compromising measurement accuracy.

The highest rate was found for 0 and 400 mM of [PB], with the minimum at about 50 mM.

After 1 h of incubation in different concentrations of PB (0, 50, and 400 mM) and the same 15‐mM Fum‐*d*_2_, microscopic analysis of yeast cells (Figure 7b,c) showed different biophysical responses:

Cells in 0‐mM PB (hypotonic environment; *n* = 288) displayed a significantly larger mean size (*p* < 0.0001) compared with those in 50‐ or 400‐mM PB, indicating water‐influx‐induced cellular swelling was mitigated by the rigid β‐1,3‐glucan/chitin yeast cell wall. This creates high turgor pressure (usually 0.3–0.8 MPa in yeast) but did not cause bursting due to the cell wall's mechanical resilience. In contrast, cells in 400‐mM PB (hypertonic environment; *n* = 367) displayed reduced size due to water efflux and turgor loss, thereby activating the high‐osmolarity glycerol (HOG) pathway within minutes via HOG1 phosphorylation [101], and this signaling cascade initiates gluconeogenesis and generates precursors like dihydroxyacetone phosphate (DHAP) to commence glycerol biosynthesis for osmotic compensation [101, 102, 103, 104]. Cells in 50‐mM PB (nearly isotonic; *n* = 349) maintained an intermediate size with no statistically significant difference compared with 400‐mM PB (*p* ≥ 0.05), indicating minimal turgor disturbance.

The accelerated *k*_fm_ at osmotic extremes is linked to the stress responses: hypotonic conditions may alter the redox balance through mechanosensors such as Wsc1p, whereas hypertonic stress redirects NADH toward glycerol biosynthesis via transcriptional upregulation of Gpd1p/Gpp2 [101] by the HOG pathway. These preserved osmoregulatory mechanisms reflect how human cells respond to electrolyte imbalances (e.g., through NFAT5 activation during dehydration or hyponatremia), despite the absence of a protective cell wall.

DMRS could detect such metabolic adaptations that happen when osmotic stress is present. It is a noninvasive means to monitor stress‐induced shifts in redox and NADH–linked pathways, potentially enabling early detection of systemic imbalances such as those occurring in sepsis or sodium disorders [105].

### 3.8 Pyruvate‐Induced Altered Metabolism of Glucose in Yeast

Previously, using ^13^C DNP, it was demonstrated that the addition of pyruvate as a carbon source could significantly alter non‐engineered yeast metabolism without affecting the rate of glucose conversion [68, 81].

In yeast, the majority of pyruvate is converted into ethanol through alcoholic fermentation, whereas a smaller portion is utilized in respiration and other pathways [106]. Pyruvate is a more oxidized metabolite than glucose, and its metabolic fate is strongly influenced by the cellular redox state [107, 108]. This reliance can result in significant changes in metabolic pathways, enabling us to detect potential deviations from normal fermentation processes.

To confirm that, we set up a DMRS experiment where we varied concentrations of sodium pyruvate (10, 50, 100, 200, 300, 400, and 500 mM) added to yeast 10 min before the experiment. The subsequent part of the experiment was the same as before; we added different amounts of Glc*‐d*_2_ at different concentrations, which resulted in a final concentration of 15, 25, and 100 mM. The addition of glucose solution resulted in the dilution of the sodium pyruvate by a factor of 2, resulting in final concentrations of 5, 25, 50, 100, 150, 200, and 250 mM.

When we used a low concentration of Glc*‐d*_2_ (24 and 20 mM; Figures 1 and 2), no significant signals from other metabolites were visible except for ethanol. Although acetate was also present, its signal was still too low.

With an excess amount of Glc*‐d*_2_ (100 mM) alone (Figure 8b), signals from Eth‐*d*_2_, [4,4‐^2^H_2_]glutamate (Glut‐*d*_2_), [2,3‐^2^H_2_]succinate (Suc‐*d*_2_), and Ac‐*d*_2_ were found. The observation of two more metabolites is partially due to the larger amount of Glc*‐d*_2_, resulting in a higher SNR of downstream products, which coincides with previous findings, where increased production of acetate was noted when glucose levels were increased from limited to excess [109].

The addition of pyruvate resulted in an apparent decrease in the production of ethanol, acetate, and glutamate and increased production of [1,1‐^2^H_2_]acetoin (Aceto‐*d*_2_) and [1,1‐^2^H_2_]butanediol (BD‐*d*_2_) (Figures 8c–e and S1–S4). This observation does not indicate that yeast stopped producing ethanol as before; instead, ethanol production was primarily carried out from the added pyruvate (which is unlabeled and unobservable with DMRS), a downstream metabolite of glucose, rather than directly from glucose itself.

Excess pyruvate, which is highly oxidized, hindered the conversion of glucose into Glc‐*d*_2_‐derived ethanol due to reduced reductant availability (NADH/NAD^+^) [68]. This metabolic shift implies the increased accumulation of active acetaldehyde, a reaction intermediate that reacts with itself through condensation catalyzed by pyruvate decarboxylase (PDC) [110], leading to the formation of Aceto*‐d*_2_ and BD‐*d*_2_, which show deviations from the normal fermentation pattern (Figure 8a), and are almost negligible when using Glc*‐d*_2_ (Figures 1 and 8b) alone [68]. A qualitatively similar effect was observed when lower concentrations of Glc*‐d*_2_ were used (Figure S4a–f).

These results suggest that adding pyruvate to yeast cells leads to an overload in the alcoholic fermentation process, pushing the yeast cells to their limit in producing alcohol due to the additional pyruvate. Integrals of spectral lines with different amounts of glucose (15, 25, and 100 mM) along with their fitted conversion rates are provided in the Supporting Information (Figures S1a–g, S2a–g, and S3a–g and Table S2), and ^31^P spectra highlighting intracellular phosphate shifts are shown in Figure S6a–d.

### 3.9 Limitations of DMRS

Unlike ^13^C hyperpolarization techniques such as dDNP and PHIP, which require complex and expensive hardware setups, DMRS is a simple and affordable approach for metabolic studies [47].

However, DMRS is not as sensitive as ^13^C hyperpolarization [68] or scintillation counting of ^14^C decay, both of which were used for yeast cell studies [111]. Unlike ^14^C, DMRS is nonradioactive and noninvasive, has chemical resolution, and has the potential for clinical translation [47].

DMRS does not allow us to observe some important reaction intermediates, including HCO^3−^ and CO_2_; thus, we could not probe all active metabolic pathways. This is in contrast to ^13^C hyperpolarization NMR [68, 70] and partially to rapid HSQC spectroscopy [112, 113]. The other issue with DMRS is the loss of deuterium labels during metabolic processes, which affects the accuracy of metabolic flux measurements [55]. The low DMRS sensitivity does not allow the detection of numerous critical reaction intermediates with sub‐mM concentrations. Instead, only the ones that are accumulated in significant amounts, for example, we observed only fumarate convertion to malate, without subsequent metabolic steps. Coupling ^1^H NMR, DMRS, and MS is preferable for a more sensitive determination of deuterium deposition.

All experiments in the current DMRS study were performed with the 5‐mm economic NMR tube with an optimized volume of glass beads. An alternative approach would be using Shigemi tubes with a solvent‐matched thick bottom. Though they have benefits, Shigemi tubes are costly (a few hundred euros each) [114]. After use, they must be routinely cleaned, which can accidentally damage the tube. This is time‐consuming and makes them less practical for routine experiments. The economy tubes, at ~2€ per tube, can provide a 100 new tubes ready for the experiments for the price of one Shigemi tube.

Further advancements in DMRS can be realized through a multifaceted approach. Utilizing cutting‐edge probe technologies like N_2_‐cooled or HE‐cooled CryoProbe [68] and using a stronger magnetic field offers higher sensitivity and molecular resolution, facilitating more sensitive detection of deuterium‐labeled metabolites.

### 3.10 DMRS Spectral Analysis

The metabolism of NAM highlighted the difficulty of DMRS due to its inherently low spectral resolution, stemming from the low gyromagnetic ratio of ^2^H and relatively narrow chemical shift range. The kinetics (Figure 6b) were obtained from partially overlapping spectra of NAM and NA (Figure 6a) using simple integration.

Advanced spectral fitting techniques such as BATMAN [115], LCModel [116], DOLPHIN [117], and others [47] offer improved reproducibility and accuracy compared with manual analysis. Additionally, the integration of deep‐learning‐based pipelines, including convolutional neural networks (CNNs) like NMRQNet [118], streamlines the analysis process, offering unsupervised automatic identification and quantification of metabolite reaction rate with increased sensitivity.

## 4 Conclusion

We used DMRS to investigate the real‐time metabolic conversion of different deuterium‐labeled substrate molecules with yeast as a model solution. Glc*‐d*_2/7_, Pyr‐*d*_3_, Fum‐*d*_2_, Ace‐*d*_6_, and NAM‐*d*_4_ are the only compounds among the tested ones that underwent metabolic conversion. Using Fum‐*d*_2_, we found the optimal PBS buffer for the yeast cell. We demonstrated that adding pyruvate to the yeast media significantly modifies glucose metabolism while not significantly affecting NAM's metabolism.

The metabolic processes of yeast offer a streamlined and manageable framework for optimizing DMRS methodologies. The protocol and insights from our yeast studies can be directly implemented to more complex in vitro or in vivo mammalian systems, thereby bridging foundational DMRS development with preclinical and clinical metabolic imaging applications.

## Author Contributions

A.N.P. and J.B.H. led the conceptual development of the study; A.N.P. and F.A. were responsible for conducting the investigation; F.A. and C.A. conducted NMR experiments; F.A. and C.A. were responsible for data curation, DMRS experiments, analyzing data, and drafting the original manuscript; S.D. and V.A. synthesized NAM‐*d*_4_; S.K. synthesized Trp‐*d*_5_; J.D. synthesized Ala‐*d*_3_; A.B. synthesized ETX‐*d*_2_. F.H.M. and F.A. did cell cultivation and viability studies. J.B.H., M.v.G., M.A., and A.N.P. oversaw supervision and funding acquisition. All authors actively participated in discussions, contributed to the interpretation of the results, and approved the final manuscript.

## Conflicts of Interest

The authors declare no conflicts of interest.

## Supporting information

Supporting Information is available in PDF form and contains the following references [18, 19, 20, 51, 52, 98, 119, 120]. This PDF file includes Figures S1–S11 and Tables S1–S3. It provides additional information on the synthesis of Trp‐*d*_5_, Ala‐*d*_3_, NAM‐*d*_4_, and ETX‐*d*_2_. It includes additional pyruvate‐modified kinetics of Glc*‐d*_2_ and NAM‐*d*_4_ metabolism (Figures S4a–f and S5a–b, respectively), and corresponding ^31^P NMR spectra (Figure S6a–d), as well as individual ^2^H NMR spectra of 26 deuterated compounds (Figure S9a–x) as listed in Table 1, and reference spectra of PBS without/with yeast used for [HDO] calibration (Figures S10a–c and S11, respectively). A separate table for measured *T*_1_, *T*_2_, and chemical shifts of Form‐*d*, Glc‐*d*_2_, and Ser‐*d*_3_ at 293 K is provided in Table S1. Kinetics fitting parameters supporting Figure 2 are summarized in Table S3; additional rate constants are mentioned in Table S2. **TABLE S1:** nbm70121‐sup‐0001‐Supplementary_Material.docx. ^2^H NMR parameters (*T*_1_, *T*_2_, and chemical shift) for a range of a few deuterium‐labeled substrates in PBS (100 mM, pH 7.4, 293 K, and 9.4 T) if not specified otherwise. The substrate concentrations were in the range of 10–30 mM. The chemical shifts were calibrated to the residual water resonance HDO, set at 4.70 ppm. When the spectra were unresolved or contained multiple lines close to each other, the total effective relaxation time for these deuterons was also estimated, denoted as “tot.”
**TABLE S2:** Overview of estimated exchange rate constants. Exchange rates (*k*, min^−1^) of various metabolites such as [2,2‐^2^H_2_]ethanol or [2,2,2‐^2^H_3_]ethanol (Eth‐*d*_2 or 3_), [2,2‐^2^H_2_]acetate or [2,2,2‐^2^H_3_]acetate (Ac‐*d*_2 or 3_), [2,3‐^2^H_2_]malate (Mal‐*d*_2_), [1,1,1,3,3,3‐^2^H_6_]propan‐2‐ol (Prop‐*d*_6_), [2,4,5,6‐^2^H_4_]nicotinic acid (NA‐*d*_4_), [1,1‐^2^H_2_]acetoin (Aceto‐*d*_2_), and [1,1‐^2^H_2_]butanediol (BD‐*d*_2_) derived from different deuterium‐labeled substrates including [6,6′‐^2^H_2_]glucose (Glc‐*d*_2_), [3,3,3‐^2^H_3_]pyruvate (Pyr‐*d*_3_), [2,3‐^2^H_2_]fumarate (Fum‐*d*_2_), [U‐^2^H_6_]acetone (Ace‐*d*_6_), and [2,4,5,6‐^2^H_4_]nicotinamide (NAM‐*d*_4_) under varying concentrations (conc) of sodium pyruvate and PB buffer. Substrate concentrations ranged from 10 to 30 mM, whereas sodium pyruvate concentration varied from 0 to 500 mM, and PB concentration from 0 to 400 mM. The chemical shifts were calibrated to the residual water resonance, set as 4.7 ppm. Exchange rates were measured under different repetition times (TR), considering the reference *T*_1_ values provided in Table 1 of the main text.
**TABLE S3:** Reproducibility of DMRS: Estimated glucose to ethanol conversion rates (*k*_ge_) and final ethanol concentrations (*y*_0_) across five independent replicates using commercial yeast. The estimated glucose‐to‐ethanol conversion rates (*k*_ge_) and final ethanol concentrations (*y*_0_) were obtained from five independent DMRS experiments using commercial food‐grade yeast. The relative standard deviations (RSD) or coefficient of variation (CV) for *k*_ge_ and *y*_0_ are 8.5% and 4.4%, respectively, indicating high reproducibility. Each experiment was conducted under identical conditions, with 20‐mM [6,6′‐^2^H_2_]glucose (Glc‐*d*_2_) as the substrate, a PBS concentration of 50 mM, and a 6.3‐s repetition time (TR). pH measurements after each experiment remained within a narrow range.
**FIGURE S1:** DMRS spectra of [6,6′‐^2^H_2_]glucose (Glc‐*d*_2_) (15 mM) metabolism in yeast assay as a function of added sodium pyruvate to the yeast media: 10 mM (a), 50 mM (b), 100 mM (c), 200 mM (d), 300 mM (e), 400 mM (f), and 500 mM (g). The addition of sodium pyruvate clearly reduces the ethanol and acetate levels, while increasing acetoin and 2,3‐butanediol levels. Each spectrum was recorded every 201.6 s = 3.36 min: NS = 32, TR = 6.3 s. 4.7 ppm HDO resonance was used as a reference. The PBS concentration was 50 mM. Y, Y1, Y2, Y3, Y4, and Y5 regions in the spectra from (a) to (g) were assigned to HDO, Glc‐*d*_2_, [1,1‐^2^H_2_]acetoin (Aceto‐*d*_2_), [2,2‐^2^H_2_]acetate (Ac‐*d*_2_), [1,1‐^2^H_2_]butanediol (BD‐*d*_2_), and [2,2‐^2^H_2_]ethanol (Eth‐*d*_2_).
**FIGURE S2:** DMRS spectra of [6,6′‐^2^H_2_]glucose (Glc‐*d*_2_) (25 mM) metabolism in yeast assay as a function of added sodium pyruvate to the yeast media: 10 mM (a), 50 mM (b), 100 mM (c), 200 mM (d), 300 mM (e), 400 mM (f), and 500 mM (g). The addition of sodium pyruvate clearly reduces the ethanol and acetate levels, while increasing acetoin and 2,3‐butanediol levels. Each spectrum was recorded every 201.6 s = 3.36 min: NS = 32, TR = 6.3 s. 4.7 ppm HDO resonance was used as a reference. The PBS concentration was 50 mM. Y, Y1, Y2, Y3, Y4, and Y5 regions in the spectra from (a) to (g) were assigned to HDO, Glc‐*d*_2_, [1,1‐^2^H_2_]acetoin (Aceto‐*d*_2_), [2,2‐^2^H_2_]acetate (Ac‐*d*_2_), [1,1‐^2^H_2_]butanediol (BD‐*d*_2_), and [2,2‐^2^H_2_]ethanol (Eth‐*d*_2_).
**FIGURE S3:** DMRS spectra of [6,6′‐^2^H_2_]glucose (Glc‐*d*_2_) (100 mM) metabolism in yeast assay as a function of added sodium pyruvate to the yeast media: 10 mM (a), 50 mM (b), 100 mM (c), 200 mM (d), 300 mM (e), 400 mM (f), and 500 mM (g). The addition of sodium pyruvate clearly reduces the ethanol and acetate levels, while increasing acetoin and 2,3‐butanediol levels. Each spectrum was recorded every 201.6 s = 3.36 min: NS = 32, TR = 6.3 s. 4.7 ppm HDO resonance was used as a reference. The PBS concentration was 50 mM. Y, Y1, Y2, Y3, Y4, and Y5 regions in the spectra from (a) to (g) were assigned to HDO, Glc‐*d*_2_, [1,1‐^2^H_2_]acetoin (Aceto‐*d*_2_), [2,2‐^2^H_2_]acetate (Ac‐*d*_2_), [1,1‐^2^H_2_]butanediol (BD‐*d*_2_), and [2,2‐^2^H_2_]ethanol (Eth‐*d*_2_).
**FIGURE S4:** nbm70121‐sup‐0001‐Supplementary_Material.docx. ^2^H NMR spectra and concentrations of metabolic products after 3 h of incubation period as a function of added pyruvate concentration. DMRS spectra of [6,6′‐^2^H_2_]glucose (Glc‐*d*_2_) (15 mM) and Glc‐*d*_2_ (25 mM) along with sodium pyruvate (500 mM) in yeast assay recorded every 1008 s = 16.8 min: NS = 160, TR = 6.3 s. 4.7 ppm HDO resonance was used as a reference for yeast cell suspension (a). Correlation between glucose, ethanol, acetate, 2,3‐butanediol, and acetoin averaged over 183–196 min as a function of pyruvate concentration (b–f). Excess of pyruvate resulted in decreased [2,2‐^2^H_2_]ethanol (Eth‐*d*_2_) production and increased production of higher alcohols and ketones such as [1,1‐^2^H_2_]butanediol (BD‐*d*_2_) or [1,1‐^2^H_2_]acetoin (Aceto‐*d*_2_). Eth‐*d*_2_ production decreased to about 14% when 500 mM of pyruvate was added (c). [2,2‐^2^H_2_]acetate (Ac‐*d*_2_) production increased by approximately 25% (f), reaching a maximum at 200‐mM added pyruvate, and then decreased by 34% from the initial value. Concurrently, the signal from [4,4‐^2^H_2_]glutamate (Glut‐*d*_2_) (a) dropped down at the same time the signals from BD‐*d*_2_ (d) and Aceto‐*d*_2_ (e) increased.
**FIGURE S5:** Effect of the added pyruvate on [2,4,5,6‐^2^H_4_]nicotinamide (NAM‐*d*_4_) metabolism. (a) The conversion rate constant of NAM‐*d*_4_ to [2,4,5,6‐^2^H_4_]nicotinic acid (NA‐*d*_4_), derived using a mono‐exponential decay fit as a function of the added pyruvate in the yeast media. The conversion rate peaked at 0.102 ± 0.012 min^−1^ at 100‐mM pyruvate but did not increase consistently at higher concentrations of added pyruvate. No significant change in the conversion rate of NA‐*d*_4_ was observed beyond 100‐mM pyruvate. (b) The effect of varying pyruvate concentrations of NA‐*d*_4_ in yeast media at the end of the experiment (*y*_0_). The *y*_0_ values decreased by approximately 5.35% from 0 to 500‐mM pyruvate, indicating no significant change in NA‐*d*_4_ accumulation. This suggests that pyruvate does not substantially influence the conversion of NAM to NA under these experimental conditions. The indicated standard errors are the result of mono‐exponential decay fit and not from the replicate experiments.
**FIGURE S6:** The effect of the added pyruvate on ^31^P NMR spectra. ^31^P NMR spectra of pyruvate metabolism in yeast assay as a function of added pyruvate to the yeast media: 10, 50, 300, and 500 mM recorded during the first 1.5 h of incubation (a) and subsequent 1.5 h (b). (c) and (d) show magnified spectra of (a) and (b), respectively, highlighting spectral changes for chemical shift range from −9 to −13 ppm. The introduction of excess pyruvate buffered the yeast suspension, resulting in the hydrolysis of polyphosphates such as ATP and NADP, which consequently led to an increase in inorganic phosphate (*P*_*i*_) levels. Each spectrum was acquired every 5652 s = 94.2 min: NS = 944, TR = 6 s. The ATP resonance at −10.5 ppm served as an internal reference. The PBS concentration was 50 mM.
**FIGURE S7:(a)** nbm70121‐sup‐0001‐Supplementary_Material.docx. ^1^H‐NMR‐sprectrum of [D]5‐*N*‐Acetyl‐L‐tryptophan‐ethylester in CD_2_Cl_2_.
**FIGURE S7:(b)** nbm70121‐sup‐0001‐Supplementary_Material.docx. ^1^H‐NMR‐sprectrum of *N*‐Acetyl‐L‐tryptophan‐ethylester in CD_2_Cl_2_.
**FIGURE S7:(c)** nbm70121‐sup‐0001‐Supplementary_Material.docx. ^13^C‐NMR‐sprectrum of [D]5‐*N*‐Acetyl‐L‐tryptophan‐ethylester in CD_2_Cl_2_.
**FIGURE S7:(d)** nbm70121‐sup‐0001‐Supplementary_Material.docx. ^13^C‐NMR‐sprectrum of *N*‐Acetyl‐L‐tryptophan‐ethylester in CD_2_Cl_2_.
**FIGURE S7:(e)** Calculated degree of deuteration of [D]5‐*N*‐Acetyl‐L‐tryptophan‐ethylester according to HRMS‐ESI.
**FIGURE S7:(f)** nbm70121‐sup‐0001‐Supplementary_Material.docx. ^1^H‐NMR‐sprectrum of [D]5‐L‐tryptophan hydrochloride in D_2_O.
**FIGURE S7:(g)** nbm70121‐sup‐0001‐Supplementary_Material.docx. ^1^H‐NMR‐sprectrum of L‐tryptophan hydrochloride in D_2_O.
**FIGURE S7:(h)** nbm70121‐sup‐0001‐Supplementary_Material.docx. ^13^C‐NMR‐sprectrum of [D]5‐L‐tryptophan hydrochloride in D_2_O.
**FIGURE S7:(i)** nbm70121‐sup‐0001‐Supplementary_Material.docx. ^13^C‐NMR‐sprectrum of L‐tryptophan hydrochloride in D_2_O.
**FIGURE S7:(j)** Calculated degree of deuteration of [D]5‐L‐tryptophan hydrochloride according to HRMS‐ESI.
**FIGURE S8:(a)** nbm70121‐sup‐0001‐Supplementary_Material.docx. ^1^H‐NMR‐sprectrum of [D]3‐(*S*)‐2‐(1,3‐dioxoisoindolin‐2‐yl) propanoic acid in CDCl_3_.
**FIGURE S8:(b)** nbm70121‐sup‐0001‐Supplementary_Material.docx.^1^H‐NMR‐sprectrum of (*S*)‐2‐(1,3‐dioxoisoindolin‐2‐yl) propanoic acid in CDCl_3_.
**FIGURE S8:(c)** nbm70121‐sup‐0001‐Supplementary_Material.docx. ^13^C‐NMR‐sprectrum of [D]3‐(*S*)‐2‐(1,3‐dioxoisoindolin‐2‐yl) propanoic acid in CDCl_3_.
**FIGURE S8:(d)** nbm70121‐sup‐0001‐Supplementary_Material.docx. ^13^C‐NMR‐sprectrum of (*S*)‐2‐(1,3‐dioxoisoindolin‐2‐yl) propanoic acid in CDCl_3_.
**FIGURE S8:(e)** Calculated degree of deuteration of [D]3‐(*S*)‐2‐(1,3‐dioxoisoindolin‐2‐yl) propanoic acid according to HRMS‐negative ESI.
**FIGURE S8:(f)** nbm70121‐sup‐0001‐Supplementary_Material.docx. ^1^H‐NMR‐sprectrum of [D]3‐alanine in D_2_O.
**FIGURE S8:(g)** nbm70121‐sup‐0001‐Supplementary_Material.docx. ^1^H‐NMR‐sprectrum of alanine in D_2_O.
**FIGURE S8:(h)** nbm70121‐sup‐0001‐Supplementary_Material.docx. ^13^C‐NMR‐sprectrum of [D]3‐alanine in D_2_O.
**FIGURE S8:(i)** nbm70121‐sup‐0001‐Supplementary_Material.docx. ^13^C‐NMR‐sprectrum of alanine in D_2_O.
**FIGURE S8:(j)** Calculated degree of deuteration of [D]_3_‐alanine according to HRMS‐negative ESI.
**FIGURE S9:** nbm70121‐sup‐0001‐Supplementary_Material.docx. ^2^H NMR spectra of 26 deuterium‐labeled compounds in PBS (100 mM, pH 7.4, 310 K, and 9.4 T) with their respective chemical shifts (ppm) provided in the main manuscript (Table 1). These chemical shifts were calibrated according to residual water HDO resonance set at 4.70 ppm. Compounds (n), (q), and (r) were observed in yeast media instead of PBS, as they emerged as metabolites rather than substrates in the reaction.
**FIGURE S10:** DMRS spectra of PBS solution along with glass beads: Integrals (a) and SNR (b) of n.a. ^2^H water peak for NMR spectra (c), which were recorded every 140 s = 2.3 min: NS = 8, TR = 17.5 s during ~300 min. The integral fluctuations are within the SNR range. No ^2^H signal decline was observed during this experimental time. This observation highlights that the decline of the added‐D signal in some NMR experiments reported in the main text could not be explained by the lack of NMR stability during the long time of the experiment.
**FIGURE S11:** DMRS spectra of PBS solution along with glass beads and yeast recorded every 140 s = 2.3 min: NS = 8, TR = 17.5 s.

## Data Availability

All raw DMRS data needed to evaluate the conclusions in the manuscript are present in the manuscript and/or the Supporting Information. The corresponding raw data will be accessible via Zenodo data repository https://doi.org/10.5281/zenodo.13983360.
