# Supplementary material for: Probing Intracellular Yeast Metabolism With Deuterium Magnetic Resonance Spectroscopy
Source: NMR Biomed. 2025 Sep 2;38(10):e70121. doi: 10.1002/nbm.70121 (PMC12403501; doi:10.1002/nbm.70121)
Supplement: Supplementary file 1 — Supporting Information is available in PDF form and contains the following references [18, 19, 20, 51, 52, 98, 119, 120]. This PDF file includes Figures S1–S11 and Tables S1–S3. It provides additional information on the synthesis of Trp‐d 5, Ala‐d 3, NAM‐d 4, and ETX‐d 2. It includes additional pyruvate‐modified kinetics of Glc‐d 2 and NAM‐d 4 metabolism (Figures S4a–f and S5a–b, respectively), and corresponding 31P NMR spectra (Figure S6a–d), as well as individual 2H NMR spectra of 26 deuterated compounds (Figure S9a–x) as listed in Table 1, and reference spectra of PBS without/with yeast used for [HDO] calibration (Figures S10a–c and S11, respectively). A separate table for measured T 1, T 2, and chemical shifts of Form‐d, Glc‐d 2, and Ser‐d 3 at 293 K is provided in Table S1. Kinetics fitting parameters supporting Figure 2 are summarized in Table S3; additional rate constants are mentioned in Table S2. TABLE S1: nbm70121‐sup‐0001‐Supplementary_Material.docx. 2H NMR parameters (T 1, T 2, and chemical shift) for a range of a few deuterium‐labeled substrates in PBS (100 mM, pH 7.4, 293 K, and 9.4 T) if not specified otherwise. The substrate concentrations were in the range of 10–30 mM. The chemical shifts were calibrated to the residual water resonance HDO, set at 4.70 ppm. When the spectra were unresolved or contained multiple lines close to each other, the total effective relaxation time for these deuterons was also estimated, denoted as “tot.” TABLE S2: Overview of estimated exchange rate constants. Exchange rates (k, min−1) of various metabolites such as [2,2‐2H2]ethanol or [2,2,2‐2H3]ethanol (Eth‐d 2 or 3), [2,2‐2H2]acetate or [2,2,2‐2H3]acetate (Ac‐d 2 or 3), [2,3‐2H2]malate (Mal‐d 2), [1,1,1,3,3,3‐2H6]propan‐2‐ol (Prop‐d 6), [2,4,5,6‐2H4]nicotinic acid (NA‐d 4), [1,1‐2H2]acetoin (Aceto‐d 2), and [1,1‐2H2]butanediol (BD‐d 2) derived from different deuterium‐labeled substrates including [6,6′‐2H2]glucose (Glc‐d 2), [3,3,3‐2H3]pyruvate (Pyr‐d 3), [2,3‐2H2]fumara [file NBM-38-e70121-s001.docx]

Supporting Information of

Probing intracellular yeast metabolism with deuterium magnetic resonance spectroscopy

Fatima Anum^1^, Charbel Assaf^1^, Farhad Haj Mohamad^1^, Maria Anikeeva^1^, Arne Brahms^2^, Jyotirmoy Dey^2^, Simon Kaltenberger^2^, Eric Beitz^3^, Lina Welz^4^, Victoria Annis^5^, Manuel van Gemmeren^2^, Simon Duckett^5^, Jan‑Bernd Hövener^1*^ and Andrey N. Pravdivtsev^1*^

^1^Section Biomedical Imaging, Molecular Imaging North Competence Center, Department of Radiology and Neuroradiology, University Medical Center Schleswig-Holstein, Kiel University, Am Botanischen Garten 14, 24118 Kiel.
^2^Otto Diels Institute of Organic Chemistry, Kiel University, Otto-Hahn-Platz 4, 24118 Kiel, Germany.
^3^Pharmaceutical Institute, Kiel University, Gutenbergstr. 76, 24118 Kiel.
^4^Institute of Clinical Molecular Biology, Department of Internal Medicine I, University Medical Center Schleswig-Holstein, Kiel University, Rosalind-Franklin-Straße 12 24105 Kiel, Germany.
^5^Centre for Hyperpolarization in Magnetic Resonance, University of York, Heslington, York, YO10 5DD, UK.

* Corresponding authors: [jan.hoevener@rad.uni-kiel.de](file:///\\\\uni-kiel.de\\files\\rad\\public\\section\\own_contributions\\papers\\2024_FA_yeast_DMI\\Text\\text8_Submission_NMR%20in%20Biomedicine\\text2_Revision\\jan.hoevener@rad.uni-kiel.de); [andrey.pravdivtsev@rad.uni-kiel.de](file:///\\\\uni-kiel.de\\files\\rad\\public\\section\\own_contributions\\papers\\2024_FA_yeast_DMI\\Text\\text8_Submission_NMR%20in%20Biomedicine\\text2_Revision\\andrey.pravdivtsev@rad.uni-kiel.de)

**Contents**

[^2^H NMR properties of deuterium-labeled molecules in PBS S3](#_Toc204594672)

[Exchange rates of various deuterium-labeled metabolites derived from various substrates under varied concentrations of sodium pyruvate and PB conditions S4](#_Toc204594673)

[Effect of sodium pyruvate on [6,6’-^2^H_2_]glucose (Glc-*d*_2_) metabolism in yeast S8](#_Toc204594674)

[15 mM Glc-*d*_2_ S8](#_Toc204594675)

[25 mM Glc-*d*_2_ S9](#_Toc204594676)

[100 mM Glc-*d*_2_ S10](#_Toc204594677)

[Influence of pyruvate levels on deuterium-labeled metabolites in yeast: DMRS spectra and concentration profiles over time S11](#_Toc204594678)

[Pyruvate-induced altered metabolism of nicotinamide in yeast S12](#_Toc204594679)

[^31^P MRS analysis of pyruvate-induced metabolic shifts in yeast S13](#_Toc204594680)

[Synthesis of four deuterium labeled compounds S14](#_Toc204594681)

[[2,4,5,6,7-^2^H_5_]tryptophan (Trp-*d*_5_) synthesis S14](#_Toc204594682)

[[3,3,3-^2^H_3_]alanine (Ala-*d*_3_) synthesis S22](#_Toc204594683)

[[2,4,5,6-^2^H_4_]nicotinamide (NAM-*d*_4_) synthesis S30](#_Toc204594684)

[[4,4’-^2^H_2_]ethosuximide (ETX-*d*_2_) synthesis S32](#_Toc204594685)

[^2^H NMR spectra of 26 deuterium-labeled biomolecules, drugs, metabolites, and solvents at 9.4 T and 310 K S33](#_Toc204594686)

[^2^H NMR spectra used for HDO concentration calibration S36](#_Toc204594687)

# ^2^H NMR properties of deuterium-labeled molecules in PBS

**TABLE S1.** ^2^H NMR parameters (T_1_, T_2_, and chemical shift) for a range of a few deuterium-labeled substrates in PBS (100 mM, pH 7.4, 293 K, and 9.4 T) if not specified otherwise. The substrate concentrations were in the range of 10 – 30 mM. The chemical shifts were calibrated to the residual water resonance HDO, set at 4.70 ppm. When the spectra were unresolved or contained multiple lines close to each other, the total effective relaxation time for these deuterons were also estimated, denoted as “tot”.

| **#** | **Tracers** | **Abbreviation used** | **Chemical shift, ppm** | **T_1_, ms** | **T_2_, ms** |
| --- | --- | --- | --- | --- | --- |
| **1** | **[1-^2^H]formate** | Form-*d* | 8.28 [CD] | 1390 ± 50 [CD] | 1400 ± 100 [CD] |
| **2** | **[6,6'-^2^H_2_]glucose** | Glc-*d*_2_ | 3.844 [CD(6)], 3.975 [CD(6’)] | 93±5 [tot] | 80 ± 3 [tot] |
| **3** | **[2,3,3’-^2^H_3_]serine** | Ser-*d*_3_ | 3.63 [CD(2)],  3.765, [CD(3)],  3.73 [CD(3’)], | 12±2 [CD(2)],  114±2 [CD(3,3’)], 116±2 [tot] | 117±3 [CD(2)],  109±2 [CD(3,3’)],  112±1 [tot] |

# Exchange rates of various deuterium-labeled metabolites derived from various substrates under varied concentrations of sodium pyruvate and PB conditions

**TABLE S2.** Overview of estimated exchange rate constants. Exchange rates (*k*, min^-1^) of various metabolites such as [2,2-^2^H_2_]ethanol or [2,2,2-^2^H_3_]ethanol (Eth-*d*_2 or 3_), [2,2-^2^H_2_]acetate or [2,2,2-^2^H_3_]acetate (Ac- *d*_2 or 3_), [2,3-^2^H_2_]malate (Mal-*d*_2_), [1,1,1,3,3,3-^2^H_6_]propan-2-ol (Prop-*d*_6_), [2,4,5,6-^2^H_4_]nicotinic acid (NA-*d*_4_), [1,1-^2^H_2_]acetoin (Aceto-*d*_2_), and [1,1-^2^H_2_]butanediol (BD-*d*_2_) derived from different deuterium-labeled substrates including [6,6’-^2^H_2_]glucose (Glc-*d*_2_), [3,3,3-^2^H_3_]pyruvate (Pyr-*d*_3_), [2,3-^2^H_2_]fumarate (Fum-*d*_2_), [U-^2^H_6_]acetone (Ace-*d*_6_) and [2,4,5,6-^2^H_4_]nicotinamide (NAM-*d*_4_) under varying concentrations (conc) of sodium pyruvate and PB buffer. Substrate concentrations ranged from 10 – 30 mM, while sodium pyruvate concentration varied from 0 –500 mM, and PB concentration from 0 – 400 mM. The chemical shifts were calibrated to residual water resonance, set as 4.7 ppm. Exchange rates were measured under different repetition times (TR), considering the reference T_1_ values provided in Table 1 of the main text.

| **#** | **Substrate** | **TR (seconds)** | **Substrate concentration (mM)** | **Sodium pyruvate added concentration (mM)** | **PBS (conc)** | **PB (conc)** | **Metabolite-1 [conversion rate (*k*, min^-1^)]** | **Metabolite-2 [conversion rate (*k*, min^-1^)]** | **Metabolite-3 [conversion rate (*k*, min^-1^)]** | **Metabolite-4 [conversion rate (*k*, min^-1^)]** |
| --- | --- | --- | --- | --- | --- | --- | --- | --- | --- | --- |
| **1** | **Glc-*d*_2_** | 6.3 | 24 | 0 | 50 | 0 | Eth-*d*_2_ (0.0243 ± 0.0008) | Ac-*d*_2_ (0.0244 ± 0.0037) |  |  |
| **2** | **Glc-*d*_7_** | 6.3 | 19 | 0 | 50 | 0 | Eth-*d*_2_ (0.0271 ± 0.0008) | Ac-*d*_2_ (0.0131 ± 0.0052) |  |  |
| **3** | **Pyr-*d*_3_** | 15.5 | 15 | 0 | 50 | 0 | Eth-*d*_3_ (0.0274 ± 0.00065) | Ac-d_3_ (0.025 ± 0.00339 |  |  |
| **4** | **Fum-*d*_2_** | 11.5 | 22 | 0 | 50 | 0 | Mal-*d*_2_ (sum) (0.0561 ± 0.0018) |  |  |  |
| **5** | **Ace-*d*_6_** | 17.5 | 16.6 | 0 | 50 | 0 | Prop-*d*_6_ (0.0123± 0.0004) |  |  |  |
| **6** | **NAM-*d*_4_** | 2.1 | 12 | 0 | 50 | 0 | NA-*d*_4_ (0.0669 ± 0.0043) |  |  |  |
| **7** | **Glc-*d*_2_** | 6.3 | 15 | 10 | 50 | 0 | Eth-*d*_2_ (0.02186 ± 0.0011) | Ac-*d*_2_ (0.04127 ± 0.02659) | Aceto-*d*_2_ (0.003543 ±0.00651) | BD*-d*_2_ (0.03543 ± 0.00651) |
| **8** | **Glc-*d*_2_** | 6.3 | 15 | 50 | 50 | 0 | Eth-*d*_2_ (0.01291 ± 0.000969855) | Ac-*d*_2_ (0.031335 ± 0.00005643) | Aceto-*d*_2_ (0.00161 ± 0.01448 | BD-*d*_2_ (0.03007 ± 0.00507) |
| **9** | **Glc-*d*_2_** | 6.3 | 15 | 100 | 50 | 0 | Eth-*d*_2_ (0.01875 ± 0.00113) | Ac-*d*_2_ (0.00182 ± 0.00653) | Aceto-*d*_2_ (0.00113 ± 0.04601) | BD-*d*_2_ (0.03189 ± 0.0048) |
| **10** | **Glc-*d*_2_** | 6.3 | 15 | 200 | 50 | 0 | Eth-*d*_2_ (0.01198 ± 0.000836593) | Ac-*d*_2_ (0.0066 ± 0.00261) | Aceto-d_2_ (0.008246 ± 0.0143) | BD-*d*_2_ (0.01982 ± 0.00391) |
| **11** | **Glc-*d*_2_** | 6.3 | 15 | 300 | 50 | 0 | Eth-*d*_2_ (0.02978 ± 0.00207) | Ac-*d*_2_ (0.0172 ± 0.00286) | Aceto-*d*_2_ (0.010022 ± 0.03396) | BD-*d*_2_ (0.0435 ± 0.00537) |
| **12** | **Glc-*d*_2_** | 6.3 | 15 | 400 | 50 | 0 | Eth-*d*_2_ (0.01261±0.0012) | Ac-*d*_2_ (0.00591±0.00241) | Aceto-*d*_2_ (0.03201±0.00331) | BD-*d*_2_ (0.02075±0.0028) |
| **13** | **Glc-*d*_2_** | 6.3 | 15 | 500 | 50 | 0 | Eth-*d*_2_ (0.00459 ±0 .00164) | Ac-*d*_2_ (0.00671 ± 0.00393) | Aceto-*d*_2_ (0.03627 ± 0.0018) | BD-*d*_2_ (0.02399 ± 0.00198) |
| **14** | **Glc-*d*_2_** | 6.3 | 25 | 10 | 50 | 0 | Eth-*d*_2_ (0.02754 ± 0.00137) | Ac-*d*_2_ (0.02017 ± 0.0127) | Aceto-*d*_2_ (0.00123512 ± 0.00153) | BD-*d*_2_(0.0075 ± 0.00126) |
| **15** | **Glc-*d*_2_** | 6.3 | 25 | 50 | 50 | 0 | Eth-*d*_2_ (0.02288 ± 0.00124) | Ac-*d*_2_ (0.02026 ± 0.0133) | Aceto-*d*_2_ (0.001113 ± 0.00721) | BD-*d*_2_ (0.03699 ± 0.00842) |
| **16** | **Glc-*d*_2_** | 6.3 | 25 | 100 | 50 | 0 | Eth-*d*_2_ (0.01864 ± 0.00108) | Ac-*d*_2_ (0.01116 ± 0.00729) | Aceto-*d*_2_ (0.002292 ± 0.00243) | BD-*d*_2_ (0.01209 ± 0.00339) |
| **17** | **Glc-*d*_2_** | 6.3 | 25 | 200 | 50 | 0 | Eth-*d*_2_ (0.02554 ± 0.00112) | Ac-*d*_2_ (0.02062 ± 0.00511) | Aceto-*d*_2_ (0.00140522 ± 0.00132) | BD-*d*_2_ (0.01761 ± 0.00325) |
| **18** | **Glc-*d*_2_** | 6.3 | 25 | 300 | 50 | 0 | Eth-*d*_2_ (0.02528 ± 0.0014) | Ac-*d*_2_ (0.01481 ± 0.00299) | Aceto-*d*_2_ (0.012278 ± 0.05812) | BD-*d*_2_ (0.02866 ± 0.00402) |
| **19** | **Glc-*d*_2_** | 6.3 | 25 | 400 | 50 | 0 | Eth-*d*_2_ (0.01343 ± 0.00093766) | Ac-*d*_2_ (0.0156 ± 0.00258) | Aceto-*d*_2_ (0.02482 ± 0.00259) | BD-*d*_2_ (0.02104 ± 0.00153) |
| **20** | **Glc-*d*_2_** | 6.3 | 25 | 500 | 50 | 0 | Eth-*d*_2_ (0.00942 ± 0.00124) | Ac-*d*_2_ (0.01165 ± 0.00354) | Aceto-*d*_2_ (0.02203 ± 0.00188) | BD-*d*_2_ (0.02524 ± 0.00563) |
| **21** | **Glc-*d*_2_** | 6.3 | 100 | 10 | 50 | 0 | Eth-*d*_2_ (0.014 ± 0.00153) | Ac-*d*_2_ (0.01012 ± 0.01598) | Aceto-*d*_2_ (0.002880 ± 0.00632) | BD-*d*_2_ (0.00576 ± 0.00272) |
| **22** | **Glc-*d*_2_** | 6.3 | 100 | 50 | 50 | 0 | Eth-*d*_2_ (0.01756 ± 0.00192) | Ac-*d*_2_ (0.00394 ± 0.0141) | Aceto-*d*_2_ (0.001103 ± 0.00535) | BD-*d*_2_ (0.00369 ± 0.00239) |
| **23** | **Glc-*d*_2_** | 6.3 | 100 | 100 | 50 | 0 | Eth-*d*_2_ (0.0118 ± 0.0008754) | Ac-*d*_2_ (0.00157 ± 0.00517) | Aceto-*d*_2_ (0.003281 ± 0.00133) | BD-*d*_2_ (0.00446 ± 0.00142) |
| **24** | **Glc-*d*_2_** | 6.3 | 100 | 200 | 50 | 0 | Eth-*d*_2_ (0.01233 ± 0.00142) | Ac-*d*_2_ (0.00344 ± 0.00688) | Aceto-*d*_2_ (0.00798 ± 0.00182) | BD-*d*_2_ (0.0182 ± 0.00176) |
| **25** | **Glc-*d*_2_** | 6.3 | 100 | 300 | 50 | 0 | Eth-*d*_2_ (0.01701 ± 0.000909) | Ac-*d*_2_ (0.00589 ± 0.00414) | Aceto-*d*_2_ (0.02749 ± 0.00984) | BD-*d*_2_ (0.014 ± 0.0012) |
| **26** | **Glc-*d*_2_** | 6.3 | 100 | 400 | 50 | 0 | Eth-*d*_2_ (0.01754 ± 0.00195) | Ac-*d*_2_ (0.00869 ± 0.0012) | Aceto-*d*_2_ (0.0213 ± 0.00419) | BD-*d*_2_ (0.0697 ± 0.00161) |
| **27** | **Glc-*d*_2_** | 6.3 | 100 | 500 | 50 | 0 | Eth-*d*_2_ (0.0054 ± 0.0029) | Ac-*d*_2_ (0.00994 ± 0.01031) | Aceto-*d*_2_ (0.02703 ± 0.00443) | BD-*d*_2_ (0.0511 ±0.00288) |
| **28** | **Fum-*d*_2_** | 11.5 | 15 | 0 | 0 | 0 | Mal-*d*_2_ (sum) (0.14021 ± 0.01405) |  |  |  |
| **29** | **Fum-*d*_2_** | 2.5 | 15 | 0 | 0 | 0 | Mal-*d*_2_ (sum) (0.14089 ± 0.03095) |  |  |  |
| **30** | **Fum-*d*_2_** | 2.5 | 15 | 0 | 0 | 4 | Mal-*d*_2_ (sum) (0.12194 ± 0.01856) |  |  |  |
| **31** | **Fum-*d*_2_** | 2.5 | 15 | 0 | 0 | 23 | Mal-*d*_2_ (sum) (0.1203 ± 0.00501) |  |  |  |
| **32** | **Fum-*d*_2_** | 2.5 | 15 | 0 | 0 | 27 | Mal-*d*_2_ (sum) (0.10059 ± 0.0062) |  |  |  |
| **33** | **Fum-*d*_2_** | 2.5 | 15 | 0 | 0 | 42 | Mal-*d*_2_ (sum) (0.0594 ± 0.00197) |  |  |  |
| **34** | **Fum-*d*_2_** | 11.5 | 15 | 0 | 0 | 50 | Mal-*d*_2_ (sum) (0.05605 ± 0.00175) |  |  |  |
| **35** | **Fum-*d*_2_** | 2.5 | 15 | 0 | 0 | 50 | Mal-*d*_2_ (sum) (0.05942 ± 0.00377) |  |  |  |
| **36** | **Fum-*d*_2_** | 11.5 | 15 | 0 | 0 | 100 | Mal-*d*_2_ (sum) (0.11179 ± 0.01059) |  |  |  |
| **37** | **Fum-*d*_2_** | 2.5 | 15 | 0 | 0 | 100 | Mal-*d*_2_ (sum) (0.07771 ± 0.00724) |  |  |  |
| **38** | **Fum-*d*_2_** | 2.5 | 15 | 0 | 0 | 104 | Mal-*d*_2_ (sum) (0.09325 ± 0.0034) |  |  |  |
| **39** | **Fum-*d*_2_** | 2.5 | 15 | 0 | 0 | 198 | Mal-*d*_2_ (sum) (0.31081 ± 0.0208) |  |  |  |
| **40** | **Fum-*d*_2_** | 2.5 | 15 | 0 | 0 | 209 | Mal-*d*_2_ (sum) (0.39419 ± 0.01599) |  |  |  |
| **41** | **Fum-*d*_2_** | 2.5 | 15 | 0 | 0 | 320 | Mal-*d*_2_ (sum) (0.43154 ± 0.02696) |  |  |  |
| **42** | **Fum-*d*_2_** | 2.5 | 15 | 0 | 0 | 325 | Mal-*d*_2_ (sum) (0.4448 ± 0.02083) |  |  |  |
| **43** | **Fum-*d*_2_** | 2.5 | 15 | 0 | 0 | 391 | Mal-*d*_2_ (sum) (0.48203 ± 0.03126) |  |  |  |
| **44** | **Fum-*d*_2_** | 2.5 | 15 | 0 | 0 | 400 | Mal-*d*_2_ (sum) (0.483 ± 0.03777) |  |  |  |
| **45** | **Fum-*d*_2_** | 11.5 | 15 | 0 | 0 | 400 | Mal-*d*_2_ (sum) (0.52 ± 0.0657) |  |  |  |
| **46** | **NAM-*d*_4_** | 2.1 | 10 | 10 | 50 | 0 | NA-*d*_4_ (0.05639 ± 0.0042) |  |  |  |
| **47** | **NAM-*d*_4_** | 2.1 | 10 | 50 | 50 | 0 | NA-*d*_4_ (0.06526 ± 0.00432) |  |  |  |
| **48** | **NAM-*d*_4_** | 2.1 | 10 | 100 | 50 | 0 | NA-*d*_4_ (0.10191 ± 0.01204) |  |  |  |
| **49** | **NAM-*d*_4_** | 2.1 | 10 | 200 | 50 | 0 | NA-*d*_4_ (0.05619 ± 0.00377) |  |  |  |
| **50** | **NAM-*d*_4_** | 2.1 | 10 | 300 | 50 | 0 | NA-*d*_4_ (0.05116 ± 0.00313) |  |  |  |
| **51** | **NAM-*d*_4_** | 2.1 | 10 | 400 | 50 | 0 | NA-*d*_4_ (0.06971 ± 0.00432) |  |  |  |
| **52** | **NAM-*d*_4_** | 2.1 | 10 | 500 | 50 | 0 | NA-*d*_4_ (0.06875 ± 0.00436) |  |  |  |
| **53** | **NAM-*d*_4_** | 2.1 | 10 | 0 | 50 | 0 | NA-*d*_4_ (0.04392 ± 0.00238) |  |  |  |

**TABLE S3.** Reproducibility of DMRS: Estimated glucose to ethanol conversion rates (*k*_ge_) and final ethanol concentrations (*y*_0_) across 5 independent replicates using commercial yeast. The estimated glucose-to-ethanol conversion rates (*k*_ge_) and final ethanol concentrations (*y*_0_) were obtained from five independent DMRS experiments using commercial food-grade yeast. The relative standard deviations (RSD) or coefficient of variation (CV) for *k*_ge_ and *y*_0_ are 8.5% and 4.4%, respectively, indicating high reproducibility. Each experiment was conducted under identical conditions, with 20 mM [6,6'-^2^H_2_]glucose (Glc-*d*_2_) as the substrate, a PBS concentration of 50 mM, and a 6.3 second repetition time (TR). pH measurements after each experiment remained within a narrow range.

| **#** | **Substrate** | **TR (seconds)** | **Substrate concentration (mM)** | **Sodium pyruvate added concentration (mM)** | **pH measured after experiment** | **PBS (conc)** | **Glucose to ethanol conversion rate (*k*_ge_, min^-1^)]** | **Final ethanol concentration [*y*_0,_ (mM)]** |
| --- | --- | --- | --- | --- | --- | --- | --- | --- |
| **1** | **Glc-*d*_2_** | 6.3 | 20 | 0 | 5.23 | 50 | 0.02599 ± 0.00132 | 20.86056 ± 0.33969 |
| **2** | **Glc-*d*_2_** | 6.3 | 20 | 0 | 5.31 | 50 | 0.02612 ± 0.0014 | 20.42369 ± 0.35825 |
| **3** | **Glc-*d*_2_** | 6.3 | 20 | 0 | 5.45 | 50 | 0.02685 ± 0.00143 | 19.65346 ± 0.33431 |
| **4** | **Glc-*d*_2_** | 6.3 | 20 | 0 | 5.35 | 50 | 0.03115 ± 0.00145 | 21.21547 ± 0.27113 |
| **5** | **Glc-*d*_2_** | 6.3 | 20 | 0 | 5.57 | 50 | 0.03086 ± 0.00141 | 22.1471 ± 0.29228 |
|  | | Average | | | | | 0.0282 | 20.86 |
|  | | Standard deviation | | | | | 0.0024 | 0.911 |
|  | | Relative standard deviation (%) | | | | | 8.5% | 4.4% |

# Effect of sodium pyruvate on [6,6’-^2^H_2_]glucose (Glc-*d*_2_) metabolism in yeast

## 15 mM Glc-*d*_2_

**
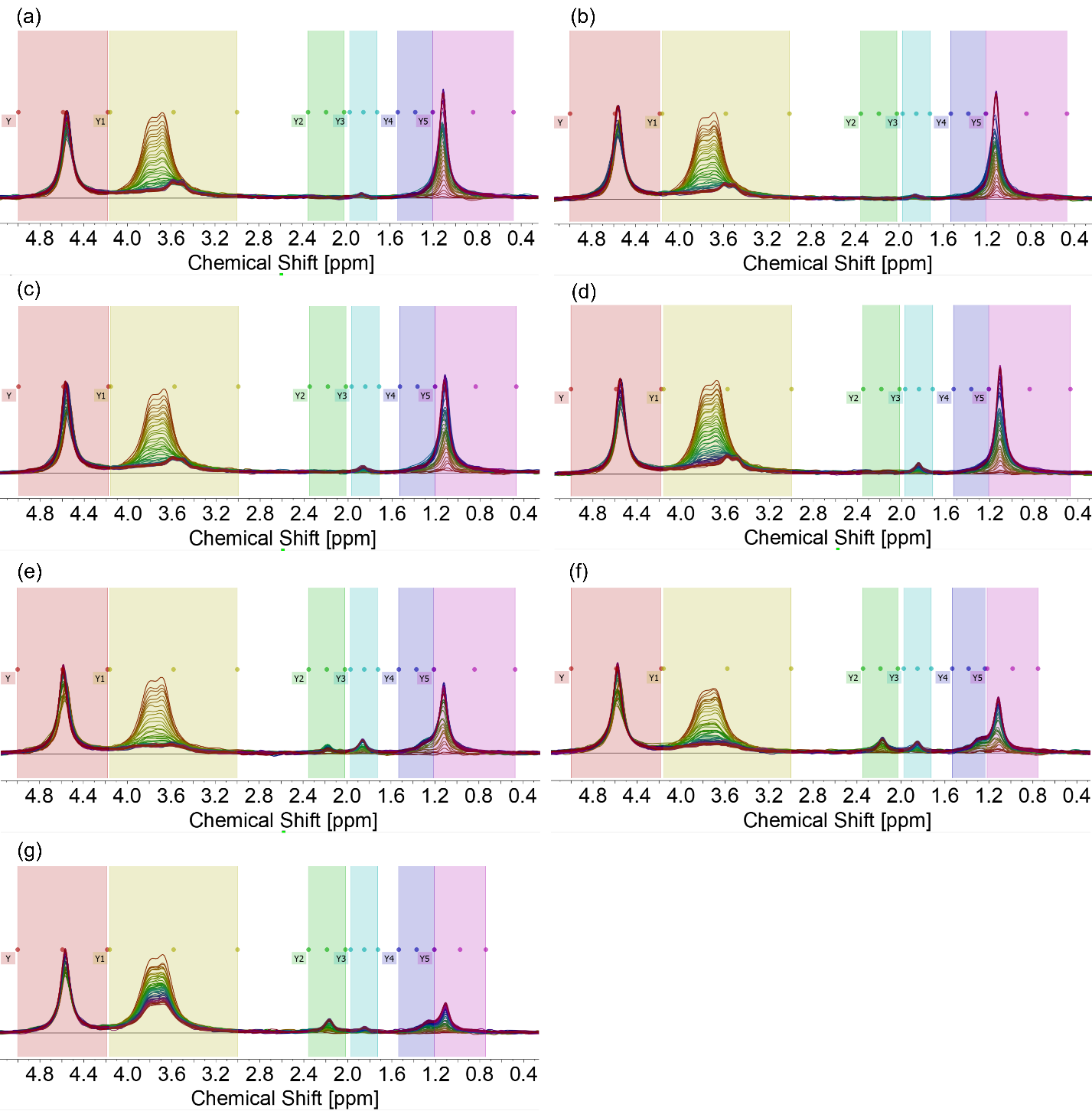
**

**FIGURE S1**. DMRS spectra of [6,6’-^2^H_2_]glucose (Glc-*d*_2_) (15 mM) metabolism in yeast assay as a function of added sodium pyruvate to the yeast media: 10 mM (a), 50 mM (b), 100 mM (c), 200 mM (d), 300 mM (e), 400 mM (f) and 500 mM (g). The addition of sodium pyruvate clearly reduces the ethanol and acetate level, while increasing acetoin and 2,3-butanediol levels. Each spectrum was recorded every 201.6 s = 3.36 min: NS = 32, TR = 6.3 s. 4.7 ppm HDO resonance was used as a reference. The PBS concentration was 50 mM. **Y, Y1, Y2, Y3, Y4 and Y5** regions in the spectra from **(a)** to **(g)** were assigned to HDO, Glc-*d*_2_, [1,1-^2^H_2_]acetoin (Aceto-*d*_2_), [2,2-^2^H_2_]acetate (Ac-*d*_2_), [1,1-^2^H_2_]butanediol (BD-*d*_2_) and [2,2-^2^H_2_]ethanol (Eth-*d*_2_).

**Note: A challenge encountered was the overlap of ethanol and 2,3-butanediol signals in the spectra, complicating their individual quantification and making it difficult to accurately assess the conversion rates of each metabolite.*

## 25 mM Glc-*d*_2_

**
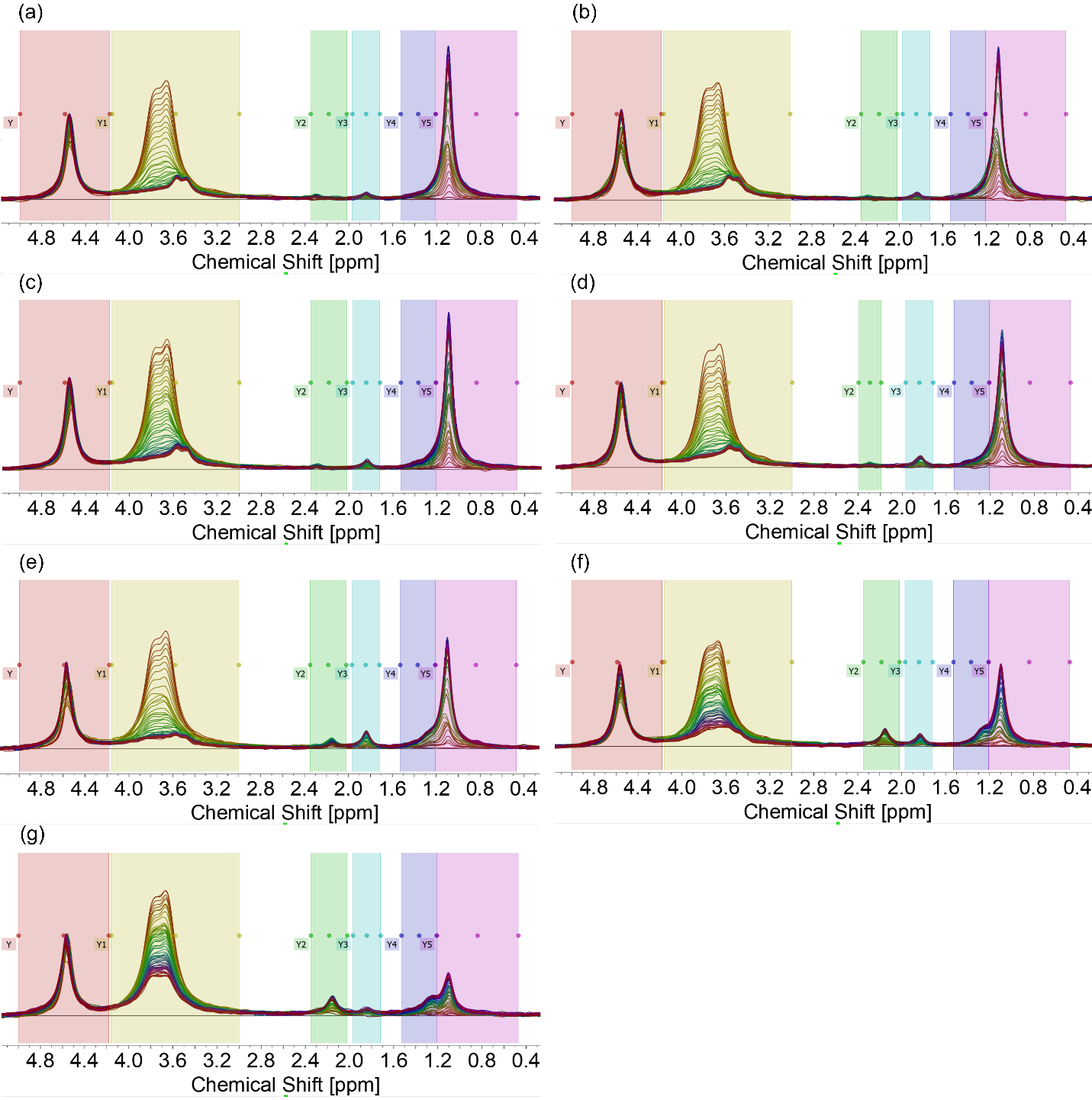
**

**FIGURE S2.** DMRS spectra of [6,6’-^2^H_2_]glucose (Glc-*d*_2_) (25 mM) metabolism in yeast assay as a function of added sodium pyruvate to the yeast media: 10 mM (a), 50 mM (b), 100 mM (c), 200 mM (d), 300 mM (e), 400 mM (f) and 500 mM (g). The addition of sodium pyruvate clearly reduces the ethanol and acetate level, while increasing acetoin and 2,3-butanediol levels. Each spectrum was recorded every 201.6 s = 3.36 min: NS = 32, TR = 6.3 s. 4.7 ppm HDO resonance was used as a reference. The PBS concentration was 50 mM. Y, Y1, Y2, Y3, Y4 and Y5 regions in the spectra from (a) to (g) were assigned to HDO, Glc-*d*_2_, [1,1-^2^H_2_]acetoin (Aceto-*d*_2_), [2,2-^2^H_2_]acetate (Ac-*d*_2_), [1,1-^2^H_2_]butanediol (BD-*d*_2_) and [2,2-^2^H_2_]ethanol (Eth-*d*_2_).

**Note: A challenge encountered was the overlap of ethanol and 2,3-butanediol signals in the spectra, complicating their individual quantification and making it difficult to accurately assess the conversion rates of each metabolite.*

## 100 mM Glc-*d*_2_

**
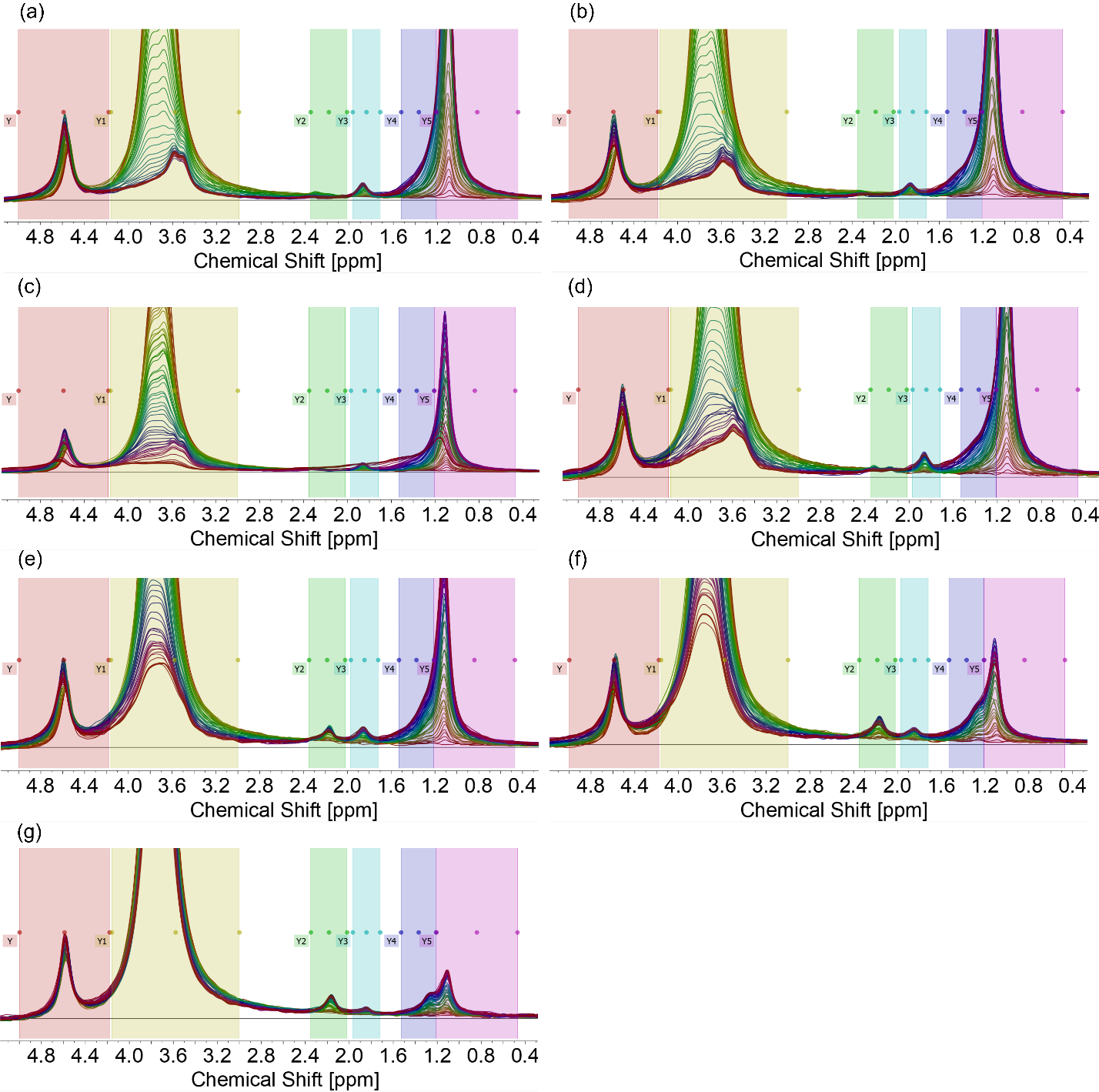
**

**FIGURE S3** DMRS spectra of [6,6’-^2^H_2_]glucose (Glc-*d*_2_) (100 mM) metabolism in yeast assay as a function of added sodium pyruvate to the yeast media: 10 mM (a), 50 mM (b), 100 mM (c), 200 mM (d), 300 mM (e), 400 mM (f) and 500 mM (g). The addition of sodium pyruvate clearly reduces the ethanol and acetate level, while increasing acetoin and 2,3-butanediol levels. Each spectrum was recorded every 201.6 s = 3.36 min: NS = 32, TR = 6.3 s. 4.7 ppm HDO resonance was used as a reference. The PBS concentration was 50 mM. Y, Y1, Y2, Y3, Y4 and Y5 regions in the spectra from (a) to (g) were assigned to HDO, Glc-*d*_2_, [1,1-^2^H_2_]acetoin (Aceto-*d*_2_), [2,2-^2^H_2_]acetate (Ac-*d*_2_), [1,1-^2^H_2_]butanediol (BD-*d*_2_) and [2,2-^2^H_2_]ethanol (Eth-*d*_2_).

**Note: A challenge encountered was the overlap of ethanol and 2,3-butanediol signals in the spectra, complicating their individual quantification and making it difficult to accurately assess the conversion rates of each metabolite.*

## Influence of pyruvate levels on deuterium-labeled metabolites in yeast: DMRS spectra and concentration profiles over time


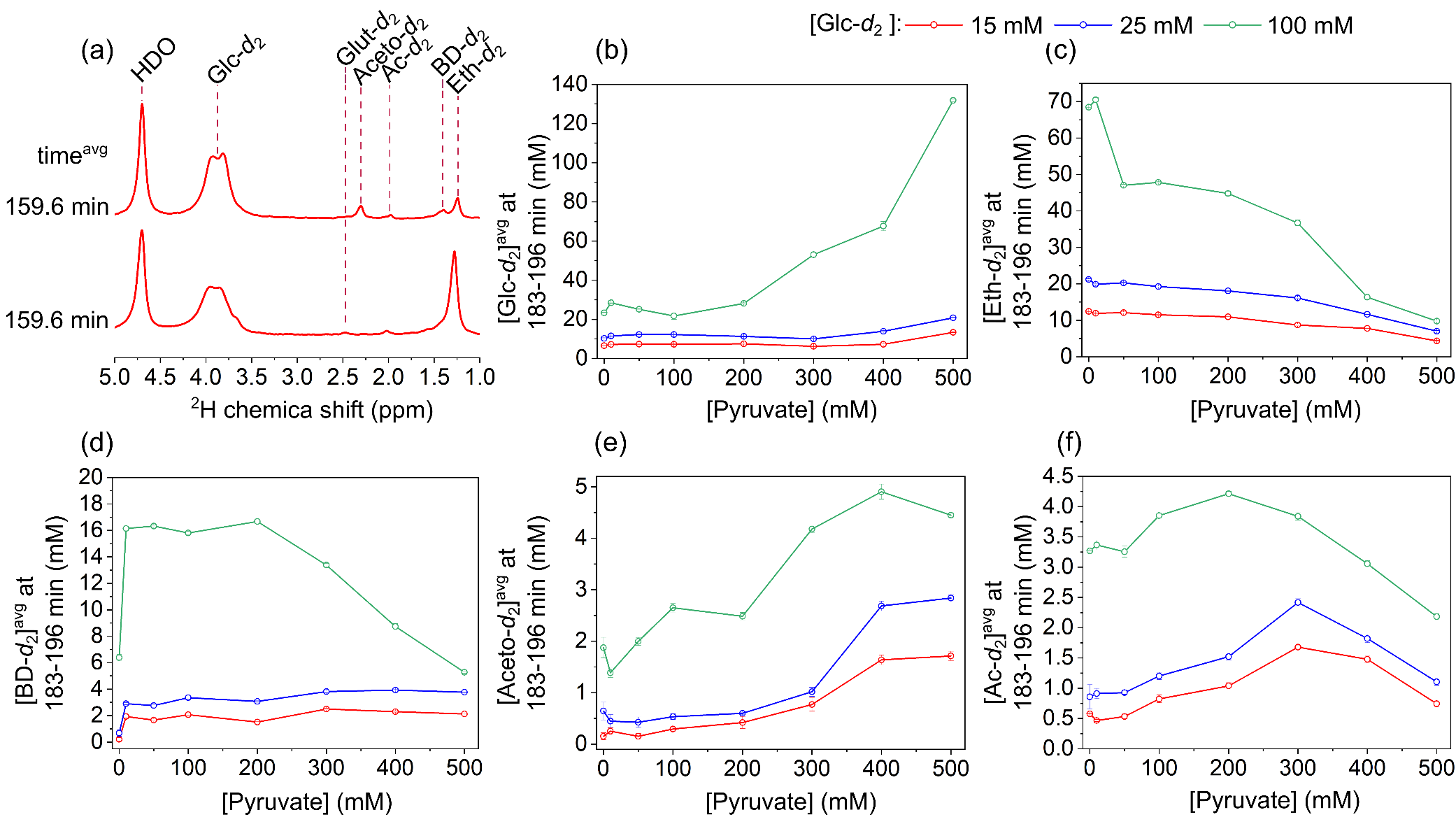


**FIGURE S4**. ^2^H NMR spectra and concentrations of metabolic products after 3 hours of incubation period as a function of added pyruvate concentration. DMRS spectra of [6,6’-^2^H_2_]glucose (Glc-*d*_2_) (15 mM) and Glc-*d*_2_ (25 mM) along with sodium pyruvate (500 mM) in yeast assay recorded every 1008 s = 16.8 min: NS = 160, TR = 6.3 s. 4.7 ppm HDO resonance was used as a reference for yeast cell suspension (a). Correlation between glucose, ethanol, acetate, 2,3-butanediol, and acetoin averaged over 183-196 mins as a function of pyruvate concentration (b-f). Excess of pyruvate resulted in decreased [2,2-^2^H_2_]ethanol (Eth-*d*_2_) production and increased production of higher alcohols and ketones such as [1,1-^2^H_2_]butanediol (BD-*d*_2_) or [1,1-^2^H_2_]acetoin (Aceto-*d*_2_).

Eth-*d*_2_ production decreased to about 14% when 500 mM of pyruvate was added (c). [2,2-^2^H_2_]acetate (Ac-*d*_2_) production increased by approximately 25% (f), reaching a maximum at 200 mM added pyruvate, and then decreased by 34% from the initial value. Concurrently, the signal from [4,4-^2^H_2_]glutamate (Glut-*d*_2_) (a) dropped down at the same time the signals from BD-*d*_2_ (d) and Aceto-*d*_2_ (e) increased.

**Note: A challenge encountered was the overlap of ethanol and 2,3-butanediol signals in the spectra, complicating their individual quantification and making it difficult to accurately assess the conversion rates of each metabolite.*

# Pyruvate-induced altered metabolism of nicotinamide in yeast

We applied the same strategy of studying pyruvate-induced metabolic alterations of [2,4,5,6-^2^H_4_]nicotinamide (NAM-*d*_4_) metabolism in yeast cells. Since pyruvate serves as the critical metabolite intermediate and is highly oxidized in nature, it can alter the redox state of the cells (Jensen *et al*. 2019, *Ref* 122). High pyruvate levels may influence the cellular redox state. However, we observed no additional signals or significant effects on the NAM to NA conversion rate. The concentrations of NA after 100 min were always within about 10% value of 0 added pyruvate value. Since all measurements were done only once without any apparent modification of metabolism, we cannot report any significant differences in NAM metabolism in the presence of pyruvate.

Pyruvate, situated at the intersection of glycolysis and the TCA cycle, influences the redox state at high concentrations but does not directly participate in the NAM salvage metabolic process. This conversion also involves the nicotinamide deamidase (Pnc1) enzyme, which are not primarily dependent on the redox state of the cells (Groth *et al*. 2021, *Ref* 100) Consequently, no significant differences were observed between conditions with or without excess pyruvate (**Table S2** and **Figure S5**).

*Method.* We used NAM-*d*_4_ at 10 mM concentration to assess the impact of added pyruvate on the kinetic control of yeast metabolism. We gathered the kinetic profile of NAM-*d*_4_ at varying concentrations of added pyruvate (0, 10, 50, 100, 200, 300, 400, and 500 mM) to the yeast incubation media.

**
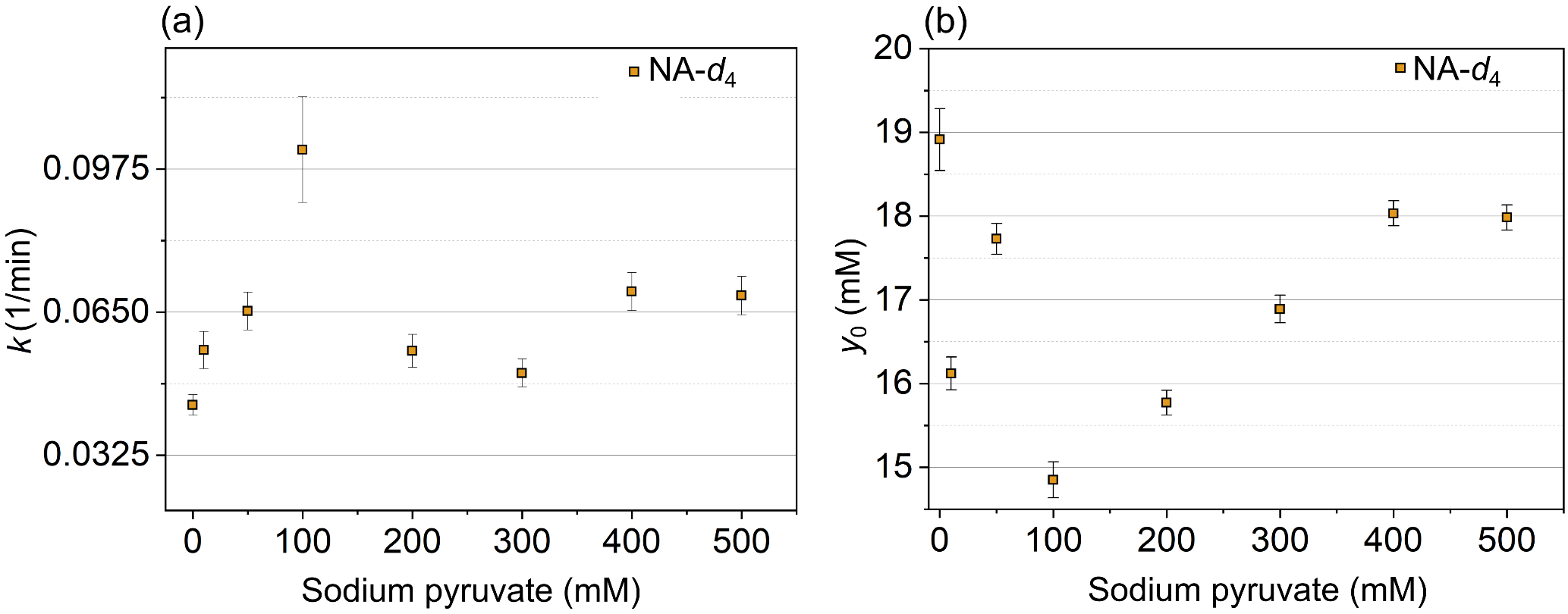
**

**FIGURE S5**. Effect of the added pyruvate on [2,4,5,6-^2^H_4_]nicotinamide (NAM-*d*_4_) metabolism. (a) The conversion rate constant of NAM-*d*_4_ to [2,4,5,6-^2^H_4_]nicotinic acid (NA-*d*_4_), derived using a mono-exponential decay fit as a function of the added pyruvate in the yeast media The conversion rate peaked at 0.102 ± 0.012 min^-1^ at 100 mM pyruvate but did not increase consistently at higher concentrations of added pyruvate. No significant change in the conversion rate of NA-*d*_4_ was observed beyond 100 mM pyruvate. (b) The effect of varying pyruvate concentrations of NA-*d*_4_ in yeast media at the end of the experiment (*y*_0_). The *y*_0_ values decreased by approximately 5.35% from 0 to 500 mM pyruvate, indicating no significant change in NA-*d*_4_ accumulation. This suggests that pyruvate does not substantially influence the conversion of NAM to NA under these experimental conditions. The indicated standard errors are the result of mono-exponential decay fit and not from the replicate experiments.

# ^31^P MRS analysis of pyruvate-induced metabolic shifts in yeast

In addition, we conducted ^31^P (phosphorus) MRS experiment, utilizing varying concentrations of pyruvate (500, 300, 50, and 10 mM) with yeast. These experiments were conducted under conditions identical to those utilized in the [6,6’-^2^H_2_]glucose (Glc-*d*₂) with excess pyruvate study to ensure comparability.

^31^P MRS is a robust technique for assessing intracellular phosphate metabolism, providing insights into the cellular energy state by detecting changes in both organic phosphates (ATP, ADP, NADP, NADPH) and inorganic phosphate (P_i_) pools (Harold *et al*. 1966, *Ref* 18), (Cassone *et al*. 1983, *Ref* 20). By monitoring these metabolites, the aim was to determine whether pyruvate alters the redox balance and energy metabolism in yeast.

The spectra obtained (**SI, Figure S6**) indicate metabolic changes. The increased amount of the added pyruvate increases the pH and observed level of ATP and ADP (**Figure S6d**). This aligns with the knowledge that high pyruvate concentrations influence the redox state. These shifts were evident in glucose metabolism but had no substantial effect on the NAM-to-NA conversion pathway. At higher pH levels, it has been reported that polyphosphate compounds such as ATP and NAD undergo hydrolysis in normally grown yeast cells. This process leads to an increase in inorganic phasphate (Pi), particularly in the 0 – 4 ppm range of the ^31^P MRS spectra (Salhany *et al*. 1975, *Ref* 19). This effect is clearly observable in the spectrum measured during the second 1.5 averaging (**Figure S6b**, compare signals between 0 and 4 with 10 and 500 mM of pyruvate concentration), where an elevated Pi signal indicates enhanced phosphate hydrolysis.


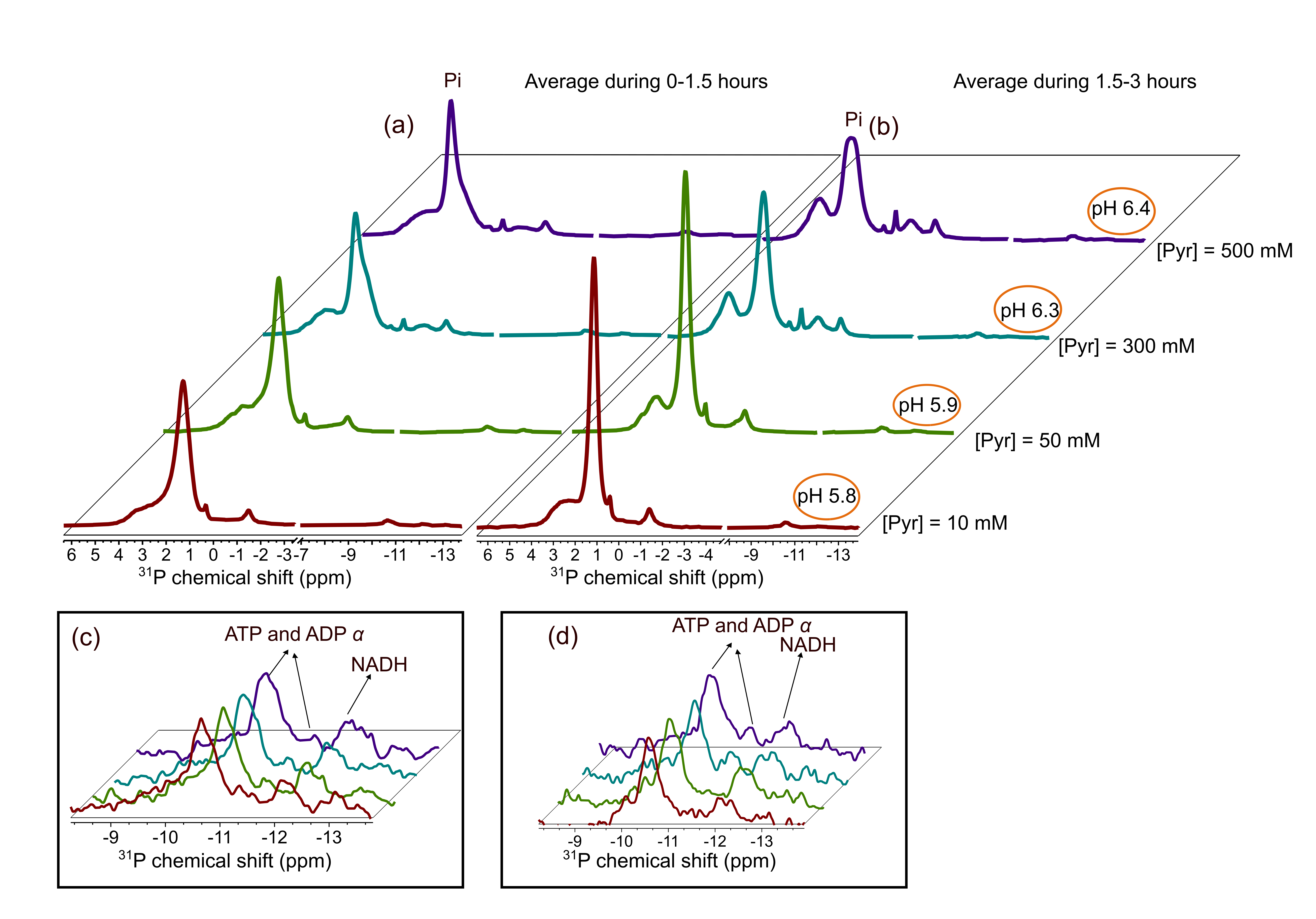


**FIGURE S6**. The effect of the added pyruvate on ^31^P NMR spectra. ^31^P NMR spectra of pyruvate metabolism in yeast assay as a function of added pyruvate to the yeast media: 10, 50, 300, and 500 mM recorded during the first 1.5 hours of incubation (a) and subsequent 1.5 hours (b). (c) and (d) show magnified spectra of (a) and (b), respectively, highlighting spectral changes for chemical shift range from -9 to -13 ppm**.** The introduction of excess pyruvate buffered the yeast suspension, resulting in the hydrolysis of polyphosphates such as ATP and NADP, which consequently led to an increase in inorganic phosphate (P_i_) levels. Each spectrum was acquired every 5652 s = 94.2 min: NS = 944, TR = 6 s. The ATP resonance at -10.5 ppm served as an internal reference. The PBS concentration was 50 mM.

# Synthesis of four deuterium labeled compounds

## [2,4,5,6,7-^2^H_5_]tryptophan (Trp-*d*_5_) synthesis

**Preparation of [D]5-*N*-Acetyl-L-tryptophan-ethylester**

This compound was prepared in analogy to a procedure from the given reference *(Dey et al.* 2024, *Ref* 52*)* An oven-dried 10 mL Schlenk-flask was charged with Pd(OAc)_2_ (1.4 mg, 0.0062 mmol, 3 mol%), 2,4,6-triisopropyl-*N*-(pyridine-2-ylmethyl)benzamide (4.1 mg, 0.012 mmol, 6 mol%), 2,5-lutidine (3.9 mg, 0.036 mmol, 18 mol%), d_1_-HFIP (0.6 mL) and a stirring bar. The solution was stirred for 20 min at room temperature. *N*-Acetyl-L-tryptophan-ethylester (54.9 mg, 0.200 mmol, 1.0 equiv) and D_2_O (1.4 mL) was added and the flask was placed in a pre-heated aluminium block at 120 °C and stirred at 1000 rpm for 18 h. Five separate reactions were performed in parallel. After the reactions have finished, the mixtures were combined in a 250 mL round-bottom flask by filtering each of them over anhydrous Na_2_SO_4_ and eluting with CH_2_Cl_2_ (40 mL each). The content of the flask was directly absorbed on silica and subjected to column chromatography (Pentane:EtOAc 70:30 → 20:80). [D]5-*N*-Acetyl-L-tryptophan-ethylester (231.9 mg, 85%) was obtained as colorless solid.

**Deuterium incorporation**: 4.87 (HRMS-ESI), 4.87 (NMR)

**NMR data of product:**

**^1^H NMR (500 MHz, CD_2_Cl_2_)** δ 8.31 (s, 1H), 7.54 (m, 0.5H = 0.95D), 7.38 (m, 0.02H = 0.98D), 7.18 (s, 0.02H = =0.98D), 7.11 – 7.09 (m, 0.02H = 0.98D), 7.02 (d, *J* = 2.5 Hz, 0.02H = 0.98D), 6.02 (d, *J* = 7.7 Hz, 1H), 4.85 (m, 1H), 4.12 (m, 2H), 3.35 – 3.23 (m, 2H), 1.92 (s, 3H), 1.23 (t, *J* = 7.1 Hz, 3H) ppm.

**^13^C NMR (126 MHz, CD_2_Cl_2_) δ** 172.3, 169.8, 136.5, 128.1, 123.3-123.1 (1C), 122.2-122.0 (1C), 119.6-119.2 (1C), 118.7-118.5 (1C), 111.4-111.2 (1C), 110.4, 61.8, 28.0, 23.4, 14.3 ppm.

**NMR data of starting material:**

**^1^H NMR (500 MHz, CD_2_Cl_2_)** δ 8.36 (br. s, 1H), 7.57 – 7.51 (m, 1H), 7.38 (dt, *J* = 8.1, 0.9 Hz, 1H), 7.18 (ddd, *J* = 8.1, 7.0, 1.2 Hz, 1H), 7.10 (ddd, *J* = 8.0, 7.0, 1.0 Hz, 1H), 7.04 – 6.99 (m, 1H), 6.03 (d, *J* = 7.8 Hz, 1H), 4.85 (m, 1H), 4.12 (m, 2H), 3.36 – 3.23 (m, 2H), 1.92 (s, 3H), 1.22 (t, *J* = 7.1 Hz, 3H) ppm.

**^13^C NMR (126 MHz, CD_2_Cl_2_)** δ 172.3, 169.8, 136.6, 128.1, 123.3, 122.5, 119.9, 118.9, 111.6, 110.6, 61.8, 28.0, 23.4, 14.3 ppm.

**FIGURE S7(a).** ^1^H-NMR-sprectrum of [D]5-*N*-Acetyl-L-tryptophan-ethylester in CD_2_Cl_2_.

**FIGURE S7(b).** ^1^H-NMR-sprectrum of *N*-Acetyl-L-tryptophan-ethylester in CD_2_Cl_2_.

**FIGURE S7(c).** ^13^C-NMR-sprectrum of [D]5-*N*-Acetyl-L-tryptophan-ethylester in CD_2_Cl_2_.

**FIGURE S7(d).** ^13^C-NMR-sprectrum of *N*-Acetyl-L-tryptophan-ethylester in CD_2_Cl_2_.


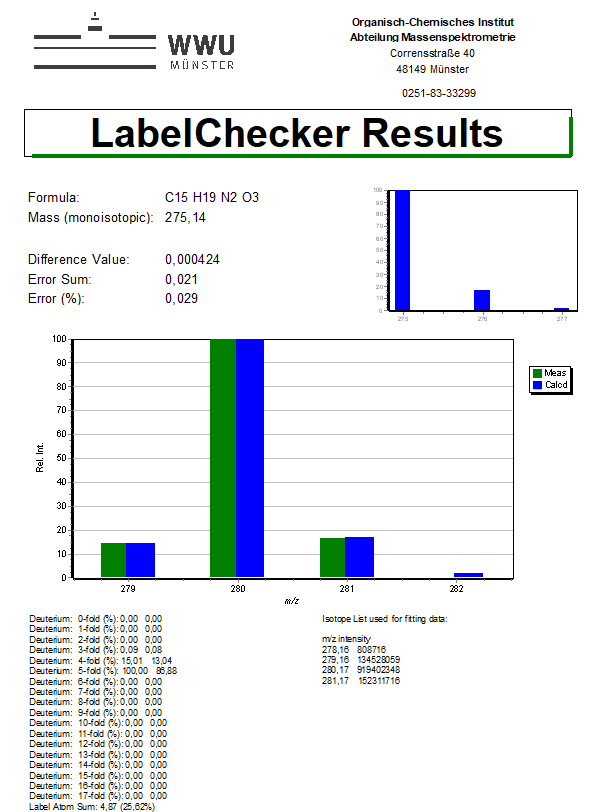


**FIGURE S7(e).** Calculated degree of deuteration of [D]5-*N*-Acetyl-L-tryptophan-ethylester according to HRMS-ESI.

**Preparation of [D]5-Tryptophan hydrochloride**


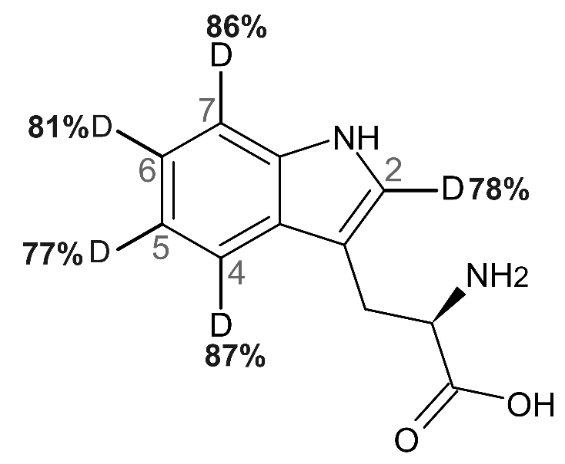


[D]5-*N*-Acetyl-L-tryptophan-ethylester (225.0 mg, 0.8202 mmol, 1.0 equiv) was added to a 100 mL round-bottom flask and a stirring bar was added. A reflux condenser was attached and the flask was evacuated and refilled with N_2_ for three times. DCl (35% in D_2_O, 1.2 mL) and D_2_O (11 mL) were added and the flask was placed in an oil-bath at 105 °C for 6h while keeping the reflux condenser attached and the apparatus under N_2_. All volatiles were removed using a rotary evaporator and the residue was further dried in high-vacuum. This procedure was repeated two more times. Afterwards H_2_O (40 mL) was added and the solution was stirred at room temperature for 6h. The solution was filtered through cotton directly into a separatory funnel and the aqueous phase was extracted with ethyl acetate (3 × 10 mL). The aqueous phase was evaporated and dried under high vacuum to afford [D]5-Tryptophan hydrochloride (140 mg, 70%) as a brown solid.

**Deuterium incorporation**: 3.96 (HRMS-ESI), 4.09 (NMR)

**NMR data of product:**

**^1^H NMR (500 MHz, D_2_O) δ** 7.72 – 7.67 (m, 0.13H = 0.87D), 7.56 – 7.47 (m, 0.14H = 0.86D), 7.33 (s, 0.22H = 0.78D), 7.31 – 7.25 (m, 0.19H = 0.81D), 7.23 – 7.12 (m, 0.23H = 0.77D), 4.32 (dd, *J* = 7.3, 5.2 Hz, 1H), 3.52 (dd, *J* = 15.4, 5.3 Hz, 1H), 3.43 (dd, *J* = 15.4, 7.4 Hz, 1H) ppm.

**^13^C NMR (126 MHz, D_2_O) δ** 175.2, 139.0, 129.2, 128.1, 125.0, 122.3-122.2 (1C), 121.0, 114.8, 109.3-109.1 (1C), 56.4, 28.6 ppm.

**NMR data of starting material:**

**^1^H NMR (500 MHz, D_2_O) δ** 7.70 (m, 1H), 7.53 (m, 1H), 7.33 (s, 1H), 7.28 (ddd, *J* = 8.2, 7.0, 1.1 Hz, 1H), 7.19 (ddd, *J* = 8.0, 7.0, 1.0 Hz, 1H), 4.34 (dd, *J* = 7.3, 5.3 Hz, 1H), 3.52 (ddd, *J* = 15.4, 5.3, 0.8 Hz, 1H), 3.43 (ddd, *J* = 15.4, 7.3, 0.7 Hz, 1H) ppm.

**^13^C NMR (126 MHz, D_2_O) δ** 175.0, 139.1, 129.3, 128.1, 125.0, 122.3, 121.0, 114.8, 109.2, 56.3, 28.6 ppm.

**FIGURE S7(f).** ^1^H-NMR-sprectrum of [D]5-L-tryptophan hydrochloride in D_2_O.

**FIGURE S7(g).** ^1^H-NMR-sprectrum of L-tryptophan hydrochloride in D_2_O.

**FIGURE S7(h).** ^13^C-NMR-sprectrum of [D]5-L-tryptophan hydrochloride in D_2_O.

**FIGURE S7(i).** ^13^C-NMR-sprectrum of L-tryptophan hydrochloride in D_2_O.


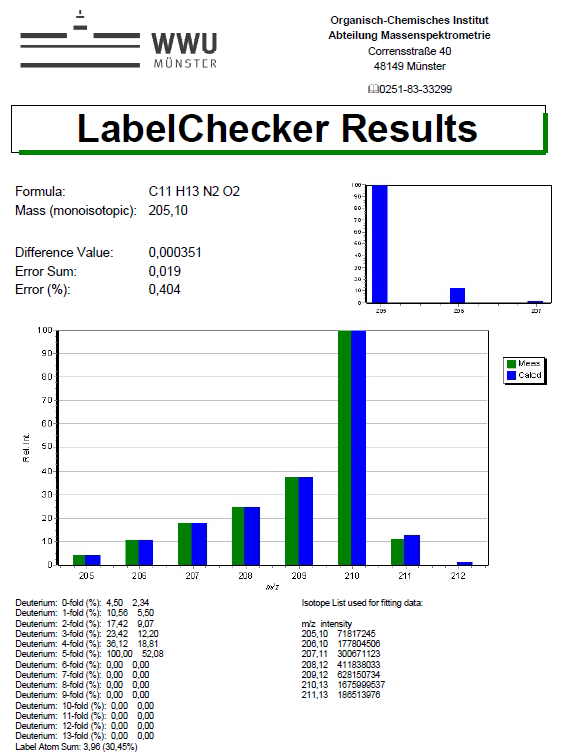


**FIGURE S7(j).** Calculated degree of deuteration of [D]5-L-tryptophan hydrochloride according to HRMS-ESI.

## [3,3,3-^2^H_3_]alanine (Ala-*d*_3_) synthesis

**Preparation of [D]3-2-(1,3-dioxoisoindolin-2-yl)propanoic acid**

Synthesized according to a procedure from the given reference (Uttrey *et al*. 2021, *Ref* 51) An oven dried air filled 10 mL Schlenk tube was charged with (*S*)-2-(1,3-dioxoisoindolin-2-yl) propanoic acid (43.8 mg, 0.200 mmol), Pd(OAc)_2_ (4.5 mg, 20 μmol, 10 mol%), *N*-(2-(dimethylamino)ethyl)-2,4,6-triisopropylbenzamide **L** (12.8 mg, 40.0 μmol, 20 mol%), Ag_2_CO_3_ (13.8 mg, 50.0 μmol, 0.25 equiv) and d_1_-HFIP (2.00 mL). The reaction mixture was stirred for 24 h at 90 °C. The mixture was allowed to cool to room temperature and formic acid (0.1 mL) was added. The mixture was transferred to a separatory funnel with CH_2_Cl_2_ (10 mL) and diluted HCl (5 wt%, 20 mL) was added. The mixture was extracted with CH_2_Cl_2_ (3 x 25 mL), the combined organic phases were dried over Na_2_SO_4_, and all volatiles were removed under reduced pressure and the target compound was prepared. Purification was achieved by silica gel column chromatography (CH_2_Cl_2_:MeOH:HCO_2_H = 99:1:0.02), giving the deuterated compound as a yellow solid (41.1 mg, 0.185 mmol, 93%).

**NMR spectra of deuterated (*S*)-2-(1,3-dioxoisoindolin-2-yl) propanoic acid:**

**Deuterium Incorporation:** 2.6 D/molecule [HRMS]; 2.46 D/molecule [^1^H-NMR].

**^1^H NMR (500 MHz, CDCl_3_)** δ 7.86 (dd, *J* = 5.4, 3.0 Hz, 2H), 7.73 (dd, *J* = 5.5, 3.0 Hz, 2H), 5.11 – 4.88 (m, 1H), 1.87 – 1.56 (m, 0.54 H = 2.46 D, 82%D) ppm.

**^13^C NMR (126 MHz, CDCl_3_)** δ 175.2, 167.3, 134.2, 131.8, 123.6, 47.1 - 47.0 (1C), 14.5 - 14.4 (1C) ppm.

**NMR spectra of (*S*)-2-(1,3-dioxoisoindolin-2-yl) propanoic acid:**

**^1^H NMR (500 MHz, CDCl_3_)** δ 11.37 (s, 1H), 7.85 (dd, *J* = 5.5, 3.0 Hz, 2H), 7.72 (dd, *J* = 5.5, 3.0 Hz, 2H), 5.03 (q, *J* = 7.4 Hz, 1H), 1.71 (d, *J* = 7.4 Hz, 3H) ppm.

**^13^C NMR (126 MHz, CDCl_3_)** δ 175.8, 167.3, 134.2, 131.8, 123.6, 47.2, 15.0 ppm.

**FIGURE S8(a).** ^1^H-NMR-sprectrum of [D]3-(*S*)-2-(1,3-dioxoisoindolin-2-yl) propanoic acid in CDCl_3_.

**FIGURE S8(b).**^1^H-NMR-sprectrum of (*S*)-2-(1,3-dioxoisoindolin-2-yl) propanoic acid in CDCl_3_.

**FIGURE S8(c).**^13^C-NMR-sprectrum of [D]3-(*S*)-2-(1,3-dioxoisoindolin-2-yl) propanoic acid in CDCl_3_.

**FIGURE S8(d).** ^13^C-NMR-sprectrum of (*S*)-2-(1,3-dioxoisoindolin-2-yl) propanoic acid in CDCl_3_.


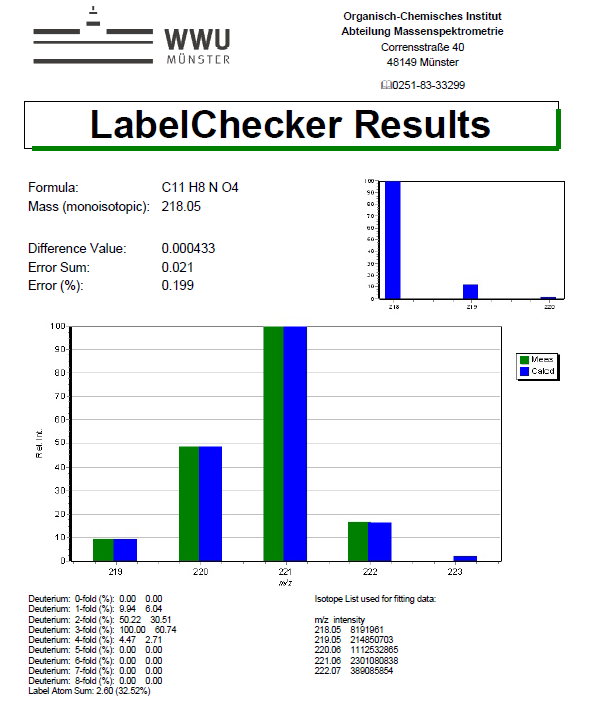


**FIGURE S8(e).** Calculated degree of deuteration of [D]3-(*S*)-2-(1,3-dioxoisoindolin-2-yl) propanoic acid according to HRMS-negative ESI.

**Preparation of [D]_3_-alanine**

Prepared in analogy to a report by the given reference (Mulzer *et al*. 1986, *Ref* 123) The deuterated (*S*)-2-(1,3-dioxoisoindolin-2-yl) propanoic acid (300 mg, 1.35 mmol) was dissolved in ethanol (5 mL) and was refluxed with an excess of hydrazine hydrate (0.2 mL, 4.05 mmol. 3 equiv.) until a colorless precipitate of phthalazone was formed. Acetone (5 mL) was added, and refluxing was continued for another 1 hr. Then the solvent was removed under reduced pressure, and the residue was diluted with water (15 mL), acidified with acetic acid to pH=4, and heated at 80 °C for 6 hrs. The crystalline precipitate was removed by filtration and the filtrate was evaporated to dryness to give deuterated Alanine (120 mg, 1.31 mmol, 97%) as a colorless solid.

**NMR spectra of the deuterated Alanine:**

**Deuterium incorporation**: 2.32 (ESI-MS), 2.46 (NMR).

**^1^H NMR (500 MHz, D_2_O)** δ 3.76 (s, 1H), 1.50 – 1.40 (m, 0.54H = 2.46 D) ppm.

**^13^C NMR (126 MHz, D_2_O)** δ 178.6, 53.1, 18.5 - 18.2 (1C) ppm.

**NMR spectra of the Alanine:**

**^1^H NMR (500 MHz, D_2_O)** δ 3.76 (q, *J* = 0.9 Hz, 1H), 1.46 (d, *J* = 0.8 Hz, 3H) ppm.

**^13^C NMR (126 MHz, D_2_O)** δ 178.5, 53.3, 18.9 ppm.

**FIGURE S8(f).** ^1^H-NMR-sprectrum of [D]3-alanine in D_2_O.

**FIGURE S8(g).** ^1^H-NMR-sprectrum of alanine in D_2_O.

**FIGURE S8(h).** ^13^C-NMR-sprectrum of [D]3-alanine in D_2_O.

**FIGURE S8(i).** ^13^C-NMR-sprectrum of alanine in D_2_O.


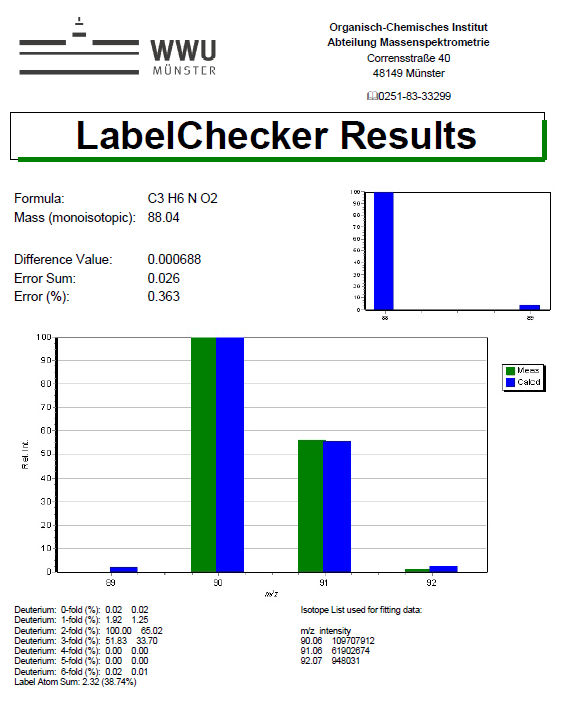


**FIGURE S8(j).** Calculated degree of deuteration of [D]_3_-alanine according to HRMS-negative ESI.

## [2,4,5,6-^2^H_4_]nicotinamide (NAM-*d*_4_) synthesis

**3-Bromo-2,4,5,6-*d*_4_-pyridine *N*-oxide**

A solution of 3-bromopyridine *N*-oxide (1.07 g, 6.15 mmol), NaOH (100 mg, 2.5 mmol, 0.4 eq.), and D_2_O (10 mL) was heated to 180 °C in a microwave for 1 h. The reaction was concentrated under reduced pressure and refreshed with ­D_2_O (10 mL), then heated to 180 °C for a further hour in a microwave. The solvent was removed under reduced pressure, and the process was repeated an additional time. The reaction was subsequently concentrated under reduced pressure and dissolved in CH_2_Cl_2_ (50 mL), dried over MgSO_4(s)_, filtered and concentrated under reduced pressure to afford 3-bromo-2,4,5,6-*d*_4_-pyridine *N*-oxide (1.05 g, 96%) as a pale yellow oil which was used without further purification, **^13^C NMR** (126 MHz, CDCl_3_) *δ* (ppm) 140.7 (t, *J* = 29.3 Hz), 137.8 (t, *J* = 29.0 Hz), 128.4 (t, *J* = 26.6 Hz), 125.6 (t, *J* = 25.7 Hz), 120.4 (s); **MS** (ESI) *m/z* 202 [(^81^Br-M + Na)^+^, 70], 200 [(^79^Br-M + Na)^+^, 72], 180 [(^81^Br-M + H)^+^, 96], 178 [(^79^Br-M + H)^+^, 100]; **HRMS** (ESI) *m/z* [M + H]^+^ calculated for C_5_H^79^BrD_4_NO 177.9800, found 177.9805 (−1.4 ppm error).

**Methyl 2,4,5,6-*d*_4_-nicotinate**

A solution of 3-bromo-2,4,5,6-*d*_4_-pyridine *N*-oxide (1.00 g, 6.17 mmol), K_2_CO_3_ (1.10 g, 8.02 mmol, 1.3eq.), PdCl_2_(dppf) (244 mg, 0.31 mmol,5 mol%) and MeOH (30 mL) was heated to 75 °C under CO_(g)_ (4 Bar) for 4 h. The reaction was allowed to cool and concentrated under reduced pressure to afford the crude product. The residue was dissolved in CH_2_Cl_2_ (30 mL), and PCl_3_ (2.0 mL) was added. Then, the reaction was heated to 40 °C for 2 h. The reaction was allowed to cool and quenched by careful addition to the ice. The resulting solution was adjusted to pH > 7 by the addition of NaHCO_3(s)_ and extracted with CH_2_Cl_2_ (3 × 50 mL). The combined extracts were concentrated under reduced pressure to afford the crude product. Purification by flash column chromatography (1:1 hexane-EtOAc) gave methyl 2,4,5,6-*d*_4_-nicotinate (644 mg, 74%) as a pale yellow solid, *R*_F_ (1:1 hexane-EtOAc) 0.3; **^1^H NMR** (400 MHz, CDCl_3_) *δ* (ppm) 3.95 (s, 3H); **^13^C NMR** (101 MHz, CDCl_3_) *δ* (ppm) 165.7 (s), 153.0( t, *J* = 27.0 Hz), 150.6 ( t, *J* = 28.5 Hz), 136.6 (t, *J* = 26.0 Hz), 125.8 (s), 122.8 (t, *J* = 24.7 Hz), 52.4 (s); **MS** (ESI) *m/z* 142 [(M + H)^+^, 100]; **HRMS** (ESI) *m/z* [M + H]^+^ calculated for C_7_H_3­_D_4_NO_2_ 142.0806, found 142.0808 (+1.6 ppm error).

**2,4,5,6-*d*_4_-Nicotinamide**

Methyl 2,4,5,6-*d*_4_-nicotinate (500 mg, 3.55 mmol) was dissolved in a methanolic solution of ammonia (30 mL of a 7 N solution in MeOH) and stirred at room temperature for 48 h. The solution was concentrated under reduced pressure to give the crude product, which was purified by reverse phase flash column chromatography (0-30% MeCN in H_2_O), gave 2,4,5,6-*d*_4_-nicotinamide (415 mg, 93%) as a white solid, **^1^H NMR** (400 MHz, *d*_6_-DMSO) *δ* (ppm) 8.17 (br s, 1H), 7.60 (br s, 1H); **^13^C NMR** (101 MHz, *d*_6_-DMSO) *δ* (ppm) 166.9 (s), 152.1 (t, *J* = 28.0 Hz), 148.8 (t, *J* = 27.0 Hz), 135.3 (t, *J* = 25.4 Hz), 130.1 (s), 123.3 (t, *J* = 25.2 Hz); **MS** (ESI) *m/z* 127 [(M + H)^+^, 100]; **HRMS** (ESI) *m/z* [M + H]^+^ calculated for C_6_H_3­_D_4_N_2_O 127.0810, found 127.0808(−1.6 ppm error).

## [4,4’-^2^H_2_]ethosuximide (ETX-*d*_2_) synthesis

**ETX-*d*_2_**

500 mg (3.54 mM) of protonated ethosuximide was added with 795 mg potassium *tert*-butanolate (7.08 mmol) and 6 mL methanol-d_4_ in a 10 mL microwave tube. The reaction was heated with 80 watts for 0.5 h in the microwave at 120°C and 8.5 bar. After cooling to room temperature, the solution was placed in a round bottle flask, and the solvent was removed under reduced pressure (50 mbar, 40 °C). The residue was resolved in 6 mL methanol-*d*_4_, transferred in a microwave tube again, and heated with 80 watts for 0.5 h at the microwave at 120°C and 8.5 bar. The solution was cooled to room temperature and slowly poured into 50 mL of 1 M aqueous HCl solution under vigorous stirring. The aqueous phase was extracted 10 times with 20 ml dichloromethane. The organic phase was dried over magnesium sulfate, and the solvent was removed under reduced pressure (20 mbar, 50°C). A low-melting white solid was obtained.

Yield 430 mg (3.00 mM) **84%**

The NMR result indicates **98%** D_2_

No traces of *tert*-butanolate were found.

# ^2^H NMR spectra of 26 deuterium-labeled biomolecules, drugs, metabolites, and solvents at 9.4 T and 310 K


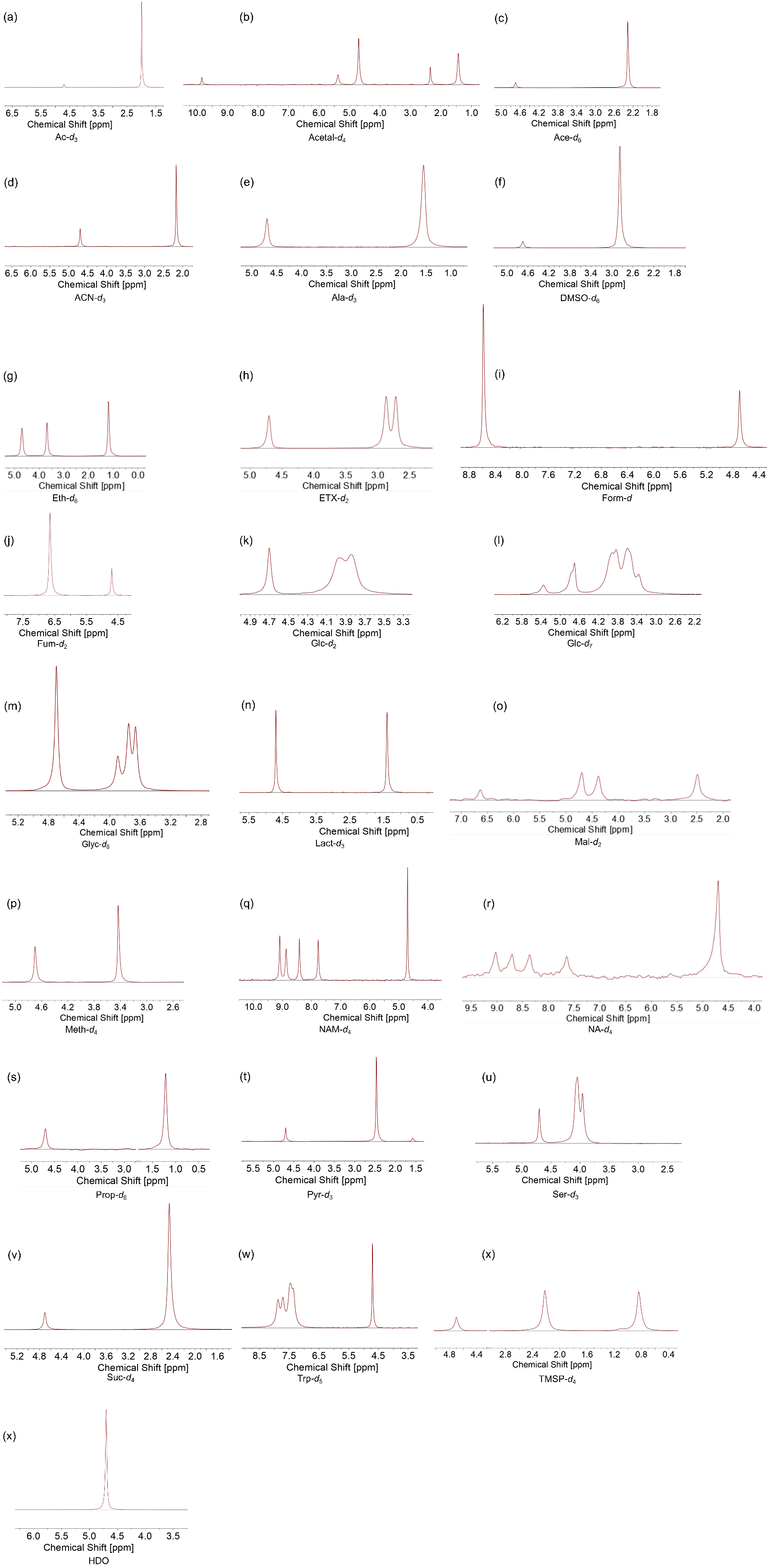


**FIGURE S9**. ^2^H NMR spectra of 26 deuterium-labeled compounds in PBS (100 mM, pH 7.4, 310 K and 9.4 T) with their respective chemical shifts (ppm) provided in the main manuscript (**Table 1**). These chemical shifts were calibrated according to residual water HDO resonance set at 4.70 ppm. *Compounds (n), (q) and (r)* were observed in yeast media instead of PBS, as they emerged as metabolites rather than substrates in the reaction.

*Note: Compound (b) Acetal-d_4_ was measured in PBS but rapidly forms its hydrated species in water (Hyd-Aceta-d_4_); thus, the spectrum represents a mixture of both forms.*

# ^2^H NMR spectra used for HDO concentration calibration


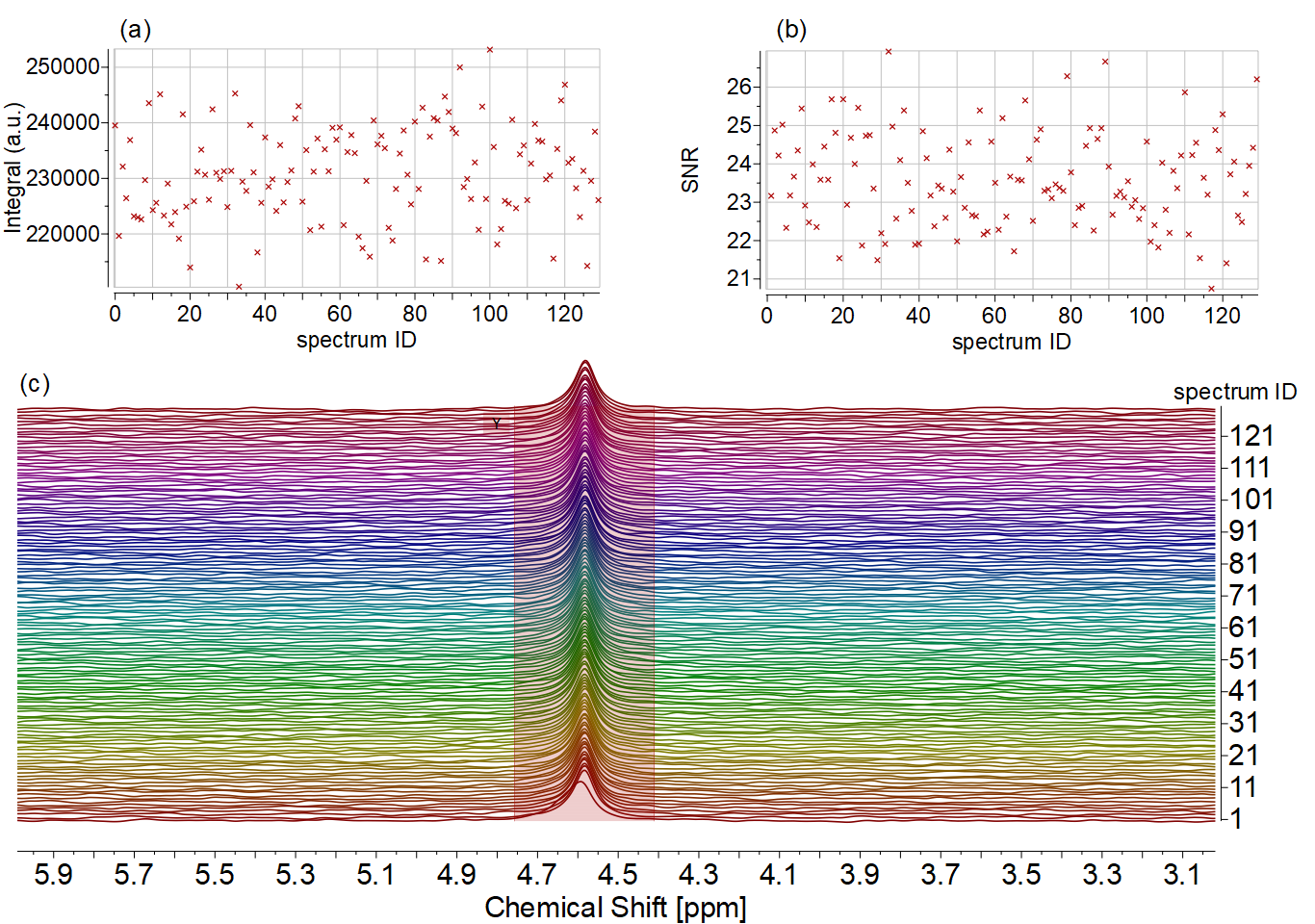


**FIGURE S10.** DMRS spectra of PBS solution along with glass beads: Integrals (a) and SNR (b) of n.a. ^2^H water peak for NMR spectra (c), which were recorded every 140 s = 2.3 min: NS= 8, TR= 17.5 s during ~300 minutes. The integral fluctuations are within the SNR range. No ^2^H signal decline was observed during this experimental time. This observation highlights that the decline of the added-D signal in some NMR experiments reported in the main text could not be explained by the lack of NMR stability during the long time of the experiment.


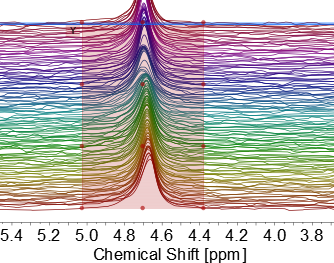


**FIGURE S11.** DMRS spectra of PBS solution along with glass beads and yeast recorded every 140 s = 2.3 min: NS= 8, TR= 17.5 s.
